# Co-Producing Narratives and Indicators as Catalysts for Adaptive Governance of a Common-Pool Resource within a Protected Area

**DOI:** 10.1007/s00267-023-01884-z

**Published:** 2023-09-23

**Authors:** Dirk J. Roux, Megan Taplin, Izak P. J. Smit, Peter Novellie, Ian Russell, Jeanne L. Nel, Stefanie Freitag, Eureta Rosenberg

**Affiliations:** 1 Scientific Services, South African National Parks, Garden Route, South Africa; 2 Sustainability Research Unit, Nelson Mandela University, George, South Africa; 3 REHABS, CNRS-Université Lyon 1-Nelson Mandela University, International Research Laboratory, George, South Africa; 4 Parks Division, South African National Parks, Knysna, South Africa; 5 Wageningen Environmental Research, Wageningen University, Wageningen, The Netherlands; 6 Environmental Learning Research Centre, Rhodes University, Makhanda, South Africa

**Keywords:** Adaptive management, Stakeholder dialogue, Governance indicators, Knysna Estuary, Principle-based framework, Social learning

## Abstract

The theory and practice of adaptive management and adaptive governance have been widely studied in the complex social contexts that mediate how humans interact with ecosystems. Adaptive governance is thought to enable adaptive management in such contexts. In this study, we examine four often-used principles of adaptive governance (polycentric institutions, collaboration, social learning and complexity thinking) to develop a framework for reflecting on adaptive governance of a social-ecological system—the Knysna Estuary in South Africa. This estuary is a priority for biodiversity conservation, as well as a common-pool resource central to livelihoods. We used the framework to structure dialogue on the extent to which the four principles of adaptive governance were being applied in the management of the Knysna Estuary. The dialogue included diverse stakeholders, from those who have the power to influence adaptive management to those most dependent on the resource for their livelihoods. Based on a combination of theory and current reality we then identified eight indicators that could be used to guide a transition towards improved adaptive governance of the estuary. These indicators were assessed and supported by most stakeholders. The main contributions of our research are (a) a process for combining theory and stakeholder dialogue to reflect on adaptive governance of a social-ecological system; (b) a set of indicators or conditions that emerged from our participatory process that can be used for reflexive monitoring and adaptation of adaptive governance of Knysna Estuary; and (c) a real-world example of seeking complementary links between adaptive governance and adaptive management to promote effective management of complex social-ecological systems.

## Introduction

Adaptive management has become a foundation of effective environmental management in contexts characterized by high levels of complexity and uncertainty (Gregory et al. 2006). Acknowledging the inevitability of imperfect understanding, adaptive management aims to combine the immediate need for management decisions with a plan for ongoing learning (Allen et al. 2011; Van Wilgen and Biggs 2011). This is done through a systematic approach of learning from management outcomes and improving resource management over time (Williams et al. 2009). Notwithstanding its intuitive ‘learning-by-doing’ appeal, a consistent message in the literature is that adaptive management is difficult to implement (Allen and Gunderson 2011) and that there are few examples of programs that have successfully applied adaptive management specifically to complex problems (Westgate et al. 2013). Implementation difficulties have prompted some scholars to suggest that adaptive management is only suitable for systems that are sufficiently controllable to allow ecological experiments (Allen et al. 2011; Rist et al. 2013) and not significantly hampered by political, social or institutional challenges (Rist et al. 2013), and where application contexts are relatively simple (e.g. single objective of concern) and small-scale (Gregory et al. 2006). This would render adaptive management of little use in many complex social-ecological systems, which characterize much of the conservation and resource-use landscape. However, an alternative view is that in complex systems, adaptive management can be facilitated by adaptive governance arrangements (e.g. Chaffin et al. 2014; Cosens and Williams 2012).

The term ‘adaptive governance’ was coined by Dietz et al. (2003) to refer to a form of environmental governance suited to situations where understanding of the linked social-ecological system is uncertain due to incomplete knowledge and contexts that may change frequently and unpredictably. As such, the focus of adaptive governance is on “flexible and learning-based collaborations and decision-making processes involving both state and non-state actors, often at multiple levels, with the aim to adaptively negotiate and coordinate management of social–ecological systems and ecosystem services across landscapes and seascapes” (Schultz et al. 2015; page 7369).

Key enablers of adaptive governance include emergent leadership, social networks that connect a variety of organizations and institutions across multiple levels and scales, and institutional learning, trust and a shared vision among stakeholders (Dietz et al. 2003; Folke et al. 2005; Karpouzoglou et al. 2016). In turn, adaptive governance promotes adaptive capacity, resolution of trade-offs, more flexible governance and resilience (an ability to absorb shocks and disturbances to retain overall function and identity) of social-ecological systems (Chaffin et al. 2014; Folke et al. 2005; Gunderson and Light 2006; Olsson et al. 2006).

The literature points to a complementary interplay between adaptive governance and adaptive management. Adaptive governance enables adaptive management by addressing the complex social contexts that mediate human interactions with ecosystems, e.g. a desired level of ecosystem functioning or access and distribution of ecosystem services (Chaffin et al. 2014). Conversely, the absence of adaptive governance can undermine adaptive management (Novellie et al. 2016). In turn, adaptive management fosters learning (through science-based experimentation and monitoring) that enables adaptive governance to take into account uncertainty in the response of ecosystems to human and management actions (Gunderson and Light 2006). Thus, while adaptive management focuses on understanding ecosystem dynamics and feeding ecological information into management organizations (Folke et al. 2005), adaptive governance provides the social networks and potential for social learning to improve the systemic uptake of new scientific knowledge generated through adaptive management as well as its integration with local knowledge, which is relevant to resource management and considered to be critical in achieving societal change (Chaffin et al. 2014).

In South Africa, the national conservation agency, South African National Parks (SANParks), has long applied a brand of adaptive management referred to as Strategic Adaptive Management (Roux and Foxcroft 2011; Roux et al. 2022). This has been done in both semi-controllable contexts, e.g. management of fire (Van Wilgen et al. 2022) and invasive alien species (Foxcroft 2009), as well as non-controllable contexts, e.g. conserving riverine biodiversity where headwaters are outside a park and managers have little direct control over their flow (Rogers and Biggs 1999; Van Wilgen and Biggs 2011; McLoughlin et al. 2021), with some success in both. Nevertheless, certain ecosystem management issues have proven particularly “wicked” (Rittel and Webber 1973), with no obvious right answers, only trade-offs between multiple and often conflicting viewpoints. The open-access Knysna Estuary in the Garden Route National Park, South Africa, is an example of such an ecosystem. Multiple role players use, impact on, and are responsible for the management of the estuary. Further, exclusion of, or control of access by, potential users is difficult and as such the estuary can be characterized as a common-pool resource (Berkes 2017; Ostrom 2008). Effective governance of common-pool resources is critical to safeguard their sustainability and promote equitable benefits to users (Berkes 2017). Here, we pose the question: what aspects or elements of adaptive governance can enable adaptive management of this common-pool resource?

We begin by outlining the social-ecological context of the Knysna estuary and present a framework that we developed for reflecting on and assessing the adaptive governance of this social-ecological system. Specifically, this framework was used to structure dialogue with diverse local and regional stakeholders on the extent to which the current governance arrangements in the Knysna Estuary conform to four commonly cited principles of adaptive governance (Dietz et al. 2003; Huitema et al. 2009; Novellie et al. 2016; Ruane 2020). The combination of theory and the current reality that emerged from the dialogue and supporting questionnaires highlighted an approach towards improved adaptive governance of the estuary, which is broadly applicable to stimulating adaptive governance of common-pool resources elsewhere.

## Case Study Description

The Knysna Estuary is located on the southern coast of South Africa, surrounded largely by the town and outlying suburbs of Knysna. The estuary is considered a national priority for biodiversity conservation and is one of only two naturally occurring estuarine bays in South Africa (Van Niekerk et al. 2019). It’s aesthetic appeal and natural resources are central to the identity and livelihoods of many of Knysna’s inhabitants. The estuary is also a popular tourist location, creating diverse direct and indirect employment and livelihood opportunities.

The topography of the estuary, surrounded by densely populated hills with direct run-off into its basin, together with the use value of the estuary, exposes it to increasing environmental pressures and threats. Settlement of people in the Knysna catchment and both recreational and resource usage are increasing. Prominent threats to the estuary include human effluent and nutrients from surrounding catchments and discharges from the local Waste Water Treatment Works (Human et al. 2016), storm-water discharges from residential and industrial areas (Hayes et al. 2022), and modification of the shoreline of much of the estuary by construction (Van Niekerk et al. 2019).

SANParks has been the designated management agency of the Knysna Estuary since 1985. In 2004, Knysna Estuary was declared a ‘Protected Environment’ (a protection category in South Africa that allows for decentralized management by local landowners), while remaining under the management authority of SANParks. More recently (in 2009), the estuary was incorporated as part of the larger Garden Route National Park, one of 21 national parks managed by SANParks. The estuary’s Protected Environment status presents an atypical situation for the management of national parks in South Africa. Most national parks are fenced with limited or controlled access, with SANParks holding a large degree of autonomy in decision making. However, the Garden Route National Park is unfenced and parts of it, for example, Knysna Estuary, are open access. Even though SANParks has designated responsibility for managing the estuary, and in reality carries most of the responsibility and autonomy for decision making, it is fundamentally dependent on governance arrangements of residents living in and around the park.

For the development of the current management plan of the Garden Route National Park (SANParks 2020), SANParks followed its standard Strategic Adaptive Management process as used for all national parks. Accordingly, a vision and high-level objectives for the park were co-produced with stakeholders (Roux et al. 2021). Thereafter, SANParks managers and scientists largely took responsibility for the more detailed breakdown of objectives into sub-objectives, actions and performance indicators. A draft management plan was made available for public comment and presented to stakeholders before being finalized. After the publication of the management plan, SANParks largely took responsibility for implementing selected actions, monitoring outcomes and evaluating the effectiveness of actions against the vision and objectives. This is similar to common adaptive management practices elsewhere (Westgate et al. 2013).

In the SANParks experience, its approach to adaptive management generally works well in relatively simple application contexts, e.g. fenced national parks where stakeholders are happy for SANParks to implement co-produced visions and objectives on their behalf. However, unfenced and partly open-access parks such as Garden Route National Park, and common-pool resources such as Knysna Estuary, with many uncontrollable variables, multiple objectives and institutional role players, overlapping governance regimes and contested user expectations, pose truly wicked problems that call for special attention to their social context (Roux et al. 2021). Although generic guidance for the management of all estuaries in the Garden Route National Park is provided in its existing management plan, no provision is made for adaptive governance. This research acknowledges the need for expanding the standard SANParks adaptive management process from one-size-fits-all to better accommodate different governance contexts, especially where there is a strong need for multi-stakeholder cooperation to achieve effective management.

## Methods

**Fig. 1:**
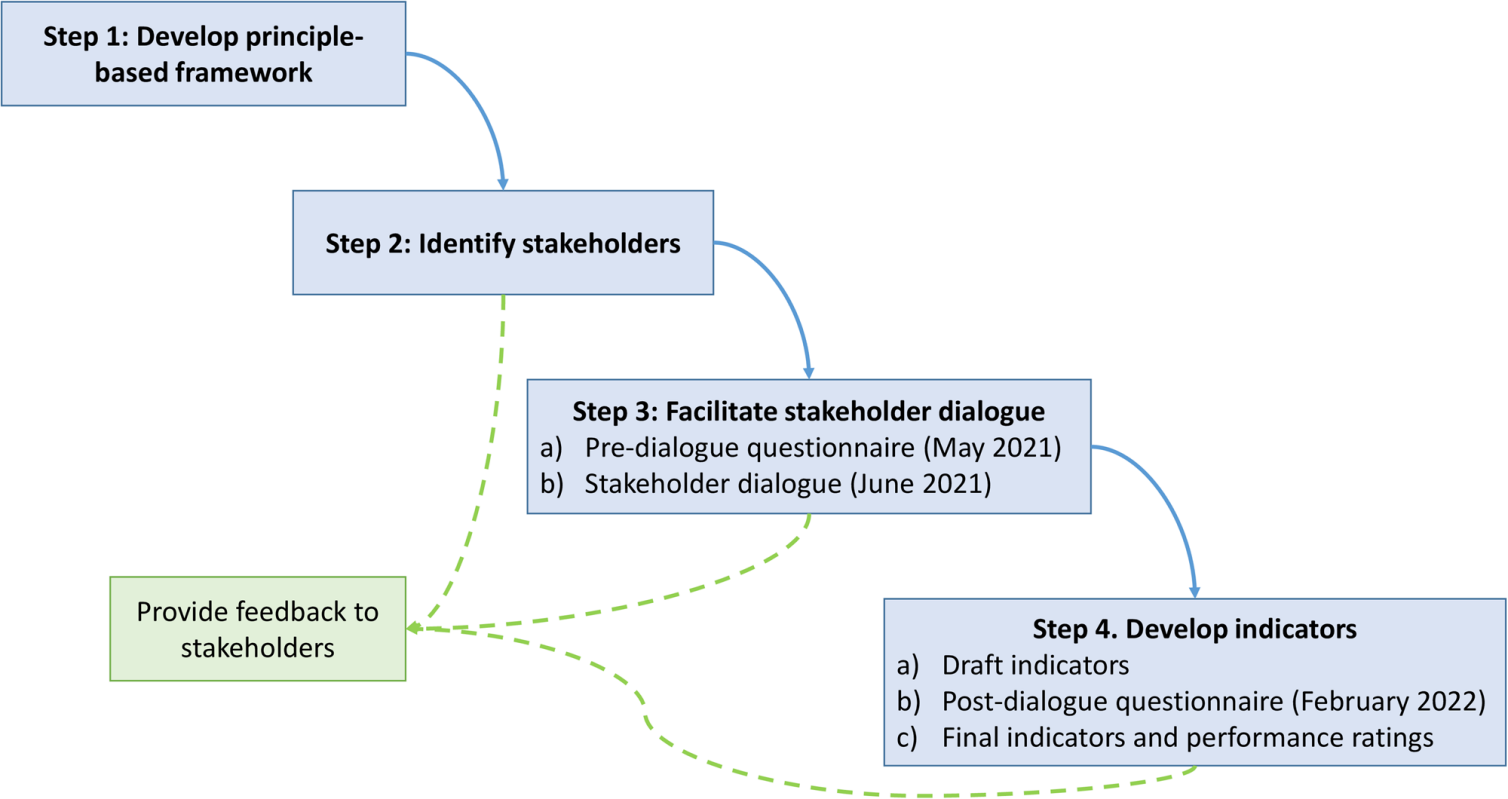
Steps of the research and stakeholder engagement processes employed in this study. Solid arrows indicate where one step provided input to a subsequent step; dotted arrows show instances where the researchers have communicated the outcome of a particular step and/or details of a subsequent step to stakeholders

The overall research approach (Fig. 1) was guided by a principle-based framework applied in a series of iterative stakeholder engagements (facilitated dialogue and questionnaires), to co-produce a set of ‘indicators’ (see sub-section on Indicators) to assess adaptive governance of the Knysna Estuary. The study took place during a time of fluctuating social distancing regulations due to the COVID-19 pandemic. As such, most of the stakeholder interactions took place online while also catering for in-person meetings to accommodate those without Internet access and who required language translation (from English to Afrikaans). This research received ethical clearance from Rhodes University (Code 1562 Jul 2020).

### Principle-Based Framework

**Fig. 2:**
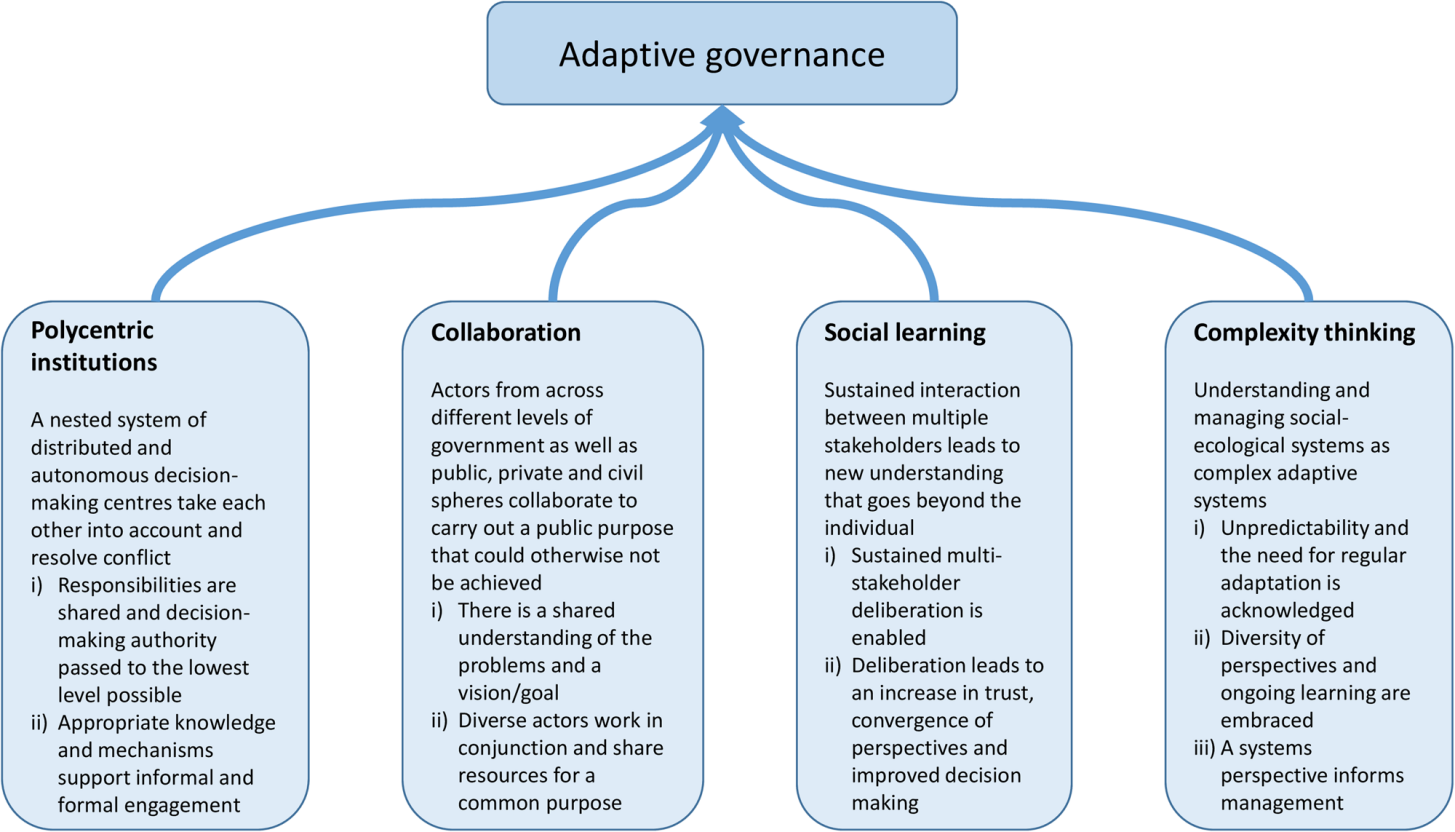
Framework for assessing and reflecting on adaptive governance of the Knysna Estuary, consisting of four principles (in bold) with associated descriptions, and the nine criteria across these (Roman numbers). Each criterion is supplemented with several questions (Supplement 1)

The stakeholders of the Knysna Estuary had little or no background in the complex concept of adaptive governance. Our approach was therefore first to develop a principle-based framework from published literature for assessing adaptive governance (Fig. 1, step 1) and then to invite stakeholders to reflect on the concept and framework. We identified four commonly-cited principles of adaptive governance: polycentric institutions, collaboration, social learning and complexity thinking (Huitema et al. 2009; Novellie et al. 2016; Ruane 2020). Next, we further explored the literature (focusing on a selection of prominent articles including seminal works and reviews) to elaborate on each principle, listing two to three criteria that related to each. Finally, we derived a set of questions that related to each criterion (Fig. 2; Supplement 1). The questions were meant to facilitate reflection by stakeholders on the degree to which each criterion/principle was being met in the governance of the Knysna Estuary.

### Stakeholder Identification

A sub-group of the authors who were familiar with the Knysna Estuary, including the SANParks manager responsible for the estuary (co-author MT), jointly identified stakeholders (Fig. 1, step 2). Stakeholder identification was guided by a set of questions proposed for achieving plurality of knowing and doing in co-production processes (Table 1; Roux et al. 2021). For each of the identified stakeholder groups, the names and contact details of one or more individuals were collated. These individuals were then invited by the SANParks manager for the Knysna Estuary to “co-reflect with SANParks on the current governance system for the Knysna Estuary”. It was explained that participation would be entirely voluntary, facilitated by people outside of SANParks, involving completing a questionnaire and taking part in a group dialogue session. Some contacts suggested additional representatives for their groups, who either represented different departments/mandates within the same organization or could provide complementary perspectives. These names were added to the list of stakeholders.

**Table 1:** Guiding questions that were used to ensure stakeholder plurality, and the resulting 21 stakeholder groups invited to participate in the questionnaires and dialogue

Questions used to guide stakeholder identification	Stakeholder groups identified
Diversity of stakeholders • Were all who hold a stake in the system identified? • Was consideration given to who will be impacted or affected by the process and its outcomes? • Was consideration given to who has the power to enable and constrain action? Diversity of perspectives • Was there an explicit attempt to empower the voices of typically marginalized groups (e.g. gender, ethnicity, age)? • Was there an explicit attempt to represent a range of geographical regions and spheres/levels (vertical as well as horizontal) of government? • Was there an explicit attempt to represent a range of knowledge (e.g. experiential, local, traditional, academic and official)? Diversity of decision-making centers and supporting roles • Was consideration given to all decision-making centers, i.e. any independent group making norms and rules (even if they are unwritten rules) within the system? • Was consideration given to those who play critical supporting roles (i.e. lack authority to make decisions but exert influence on policies or provide critical technical or financial support)?	National government • SANParks ✓ Provincial government • Western Cape Department Environmental Affairs & Development Planning ✓ • Western Cape Department Fisheries ✓ Local government • Breede-Gouritz Catchment Management Agency • Garden Route District Municipality ✓ • Knysna Municipality ✓ User groups • Fisher people* ✓ • Recreational anglers • Subsistence fishers - boat owners • Knysna Yacht Club ✓ • Leisure Island Boat Club ✓ Interest groups and NGOs • Commercial water-based business • Garden Route Biosphere Reserve • Garden Route Operators Association ✓ • Greater Knysna Business Chamber ✓ • Knysna Basin Project ✓ • Knysna Catchment Management Forum ✓ • Knysna Heads Association ✓ • Leisure Island Residents Association ✓ • Local business Research • South African Earth Observation Network ✓

A tick (✓) indicates which groups were represented in the dialogue. Responses to the two questionnaires were anonymous and represent steps 3a and 4b of Fig. 1

*This group of mostly women identify with the collective term ‘fisher people’. They collect bait and catch fish (typically with hand lines) on most days of the year and at various places along the estuary shore. They possess deep local knowledge of the estuary and fishing is central to their identity as well as an important socializing activity

### Stakeholder Dialogue

Following the initial outreach to stakeholders, 33 individuals from the 21 groups were invited to complete an online questionnaire with a mixture of open- and close-ended questions (Supplement 2). The aim of this ‘pre-dialogue questionnaire’ (Figure, step 3a;) was to (a) explain the research and its key concepts to the stakeholders, (b) obtain a better understanding of current stakeholder perceptions about the estuary and its governance within the context of the principle-based framework, and (c) provide an opportunity to anonymously and individually provide input that may be difficult in an open dialogue due to institutional mandates, power dynamics or different communication preferences. For one group (a previously marginalized group; of four individuals), manual completion of the questionnaire was facilitated. Based on this group’s language preference, the questionnaire was translated into Afrikaans. Each question was discussed in Afrikaans to ensure proper understanding. The consensus answer of the group was then captured in English on the online questionnaire form to ensure the anonymity of the results. Although four individuals from this group participated in the questionnaire, their collective answer was entered as one response as per the preference of these participants.

A facilitated dialogue was designed following Isaacs (1993) as a “collective inquiry into the processes, assumptions and uncertainties that compose everyday experiences” (Fig. 1, step 3b). Such dialogue seeks to allow greater coherence (not necessarily agreement) to emerge among a group of people, although it does not impose coherence. This requires an experienced facilitator, at least initially, who can help set up the field of inquiry and who can embody its principles and intention. By deliberately trying not to solve familiar problems in a familiar way, we aspired to open new possibilities for shared thinking. The dialogue was structured following the principles, criteria and questions laid out in the principle-based framework (Fig. 2; Supplement 1), with feedback from the pre-dialogue questionnaire (Fig. 1, step 3a) serving as context. The dialogue was facilitated to cater for hybrid online (Zoom) and in-person participation over a 3-hour period. The facilitator (co-author ER) was from a university and unknown to the participants, to help ensure the dialogue was experienced as impartial.

During the dialogue, members of the research team introduced themselves. Thereafter the objectives of the research were recapped, stating that the intention of the dialogue was for stakeholders to (a) hear each other, (b) learn from and about each other, and (c) for all to better understand the current governance of Knysna Estuary. An overview of the feedback received and initial insights from the pre-dialogue questionnaire were provided, whereafter participants were divided into four pre-determined parallel breakout groups (to promote stakeholder diversity in each group). One breakout group was held in person (in part to help empower a previously marginalized group by providing translation when necessary and ensuring their voice was being heard) and the others online. Each breakout group was allocated one ‘primary principle’ to focus their deliberation on, and, if time allowed, a ‘secondary principle’. This was done in a way so that all four principles received at least primary, and potentially secondary, attention (although not by all groups and participants). Discussion in each group was guided by a set of 7–10 prompting questions in the respective criteria of the principle-based framework. The group discussion was moderated by a member of the research team while another member captured the outcome in a way that was visible to all participating within the group. After the time allotted for breakout groups, participants re-joined in plenary and each group presented an overview of key themes that emerged from their dialogue. After each group presentation, participants from other groups could ask questions and suggest additions. Both group and plenary discussions were recorded and later transcribed.

### Indicators

Bennet and Satterfield (2018) propose that indicators be developed, in collaboration with stakeholders, to facilitate ongoing monitoring and adaptation of governance arrangements. Monitoring can be results-oriented, with an emphasis on measuring quantitative indicators as is often required for providing an accountability trail. Alternatively, monitoring can be reflexive in nature and use qualitative indicators to stimulate collective learning and change of practices (Van Mierlo et al. 2010). We believe that a reflexive framework is highly applicable to adaptive governance and here we define indicators as conditions that need to be in place to enable effective adaptive governance of the Knysna Estuary. In this sense, indicators are to provide a basis for collective and reflexive monitoring and evaluation regarding the extent to which current governance arrangements may need modification and improvement.

We developed a set of draft indicators based on the stakeholder discussions of the principle-based framework in the dialogue, in which key themes emerged from the issues, concerns and needs raised by stakeholders during the dialogue. These key themes were crafted into eight indicators of adaptive governance (two for each principle). The themes logically clustered into the eight indicators, representing a relatively comprehensible set. While the dialogue was influenced by an a priori framework, which was also debated by participants, the indicators themselves were formulated based on issues, concerns and needs that emerged from the dialogue.

The draft indicators were presented to stakeholders in a post-dialogue questionnaire (Fig. 1, step 4b; Supplement 3). Stakeholders were requested to: (a) indicate the appropriateness of each indicator for the governance of Knysna Estuary and suggest adaptation or alternatives where they did not agree, and (b) score their experience or perception regarding the current governance of Knysna Estuary on a five-point scale for each of the indicators. As in Step 3a (Fig. 1), for one group (eight individuals) completion of the post-dialogue questionnaire was facilitated by researchers capturing their responses during an in-person meeting. Results of the post-dialogue questionnaire were then used to finalize the set of indicators and develop baseline values of current performance (Fig. 1, step 4c).

## Results

### Pre-Dialogue Questionnaire

From 33 invited individuals, 27 completed questionnaires were received. The high response rate (82%) suggests that stakeholders are highly committed to the estuary and consider its governance and management important.

**Fig. 3:**
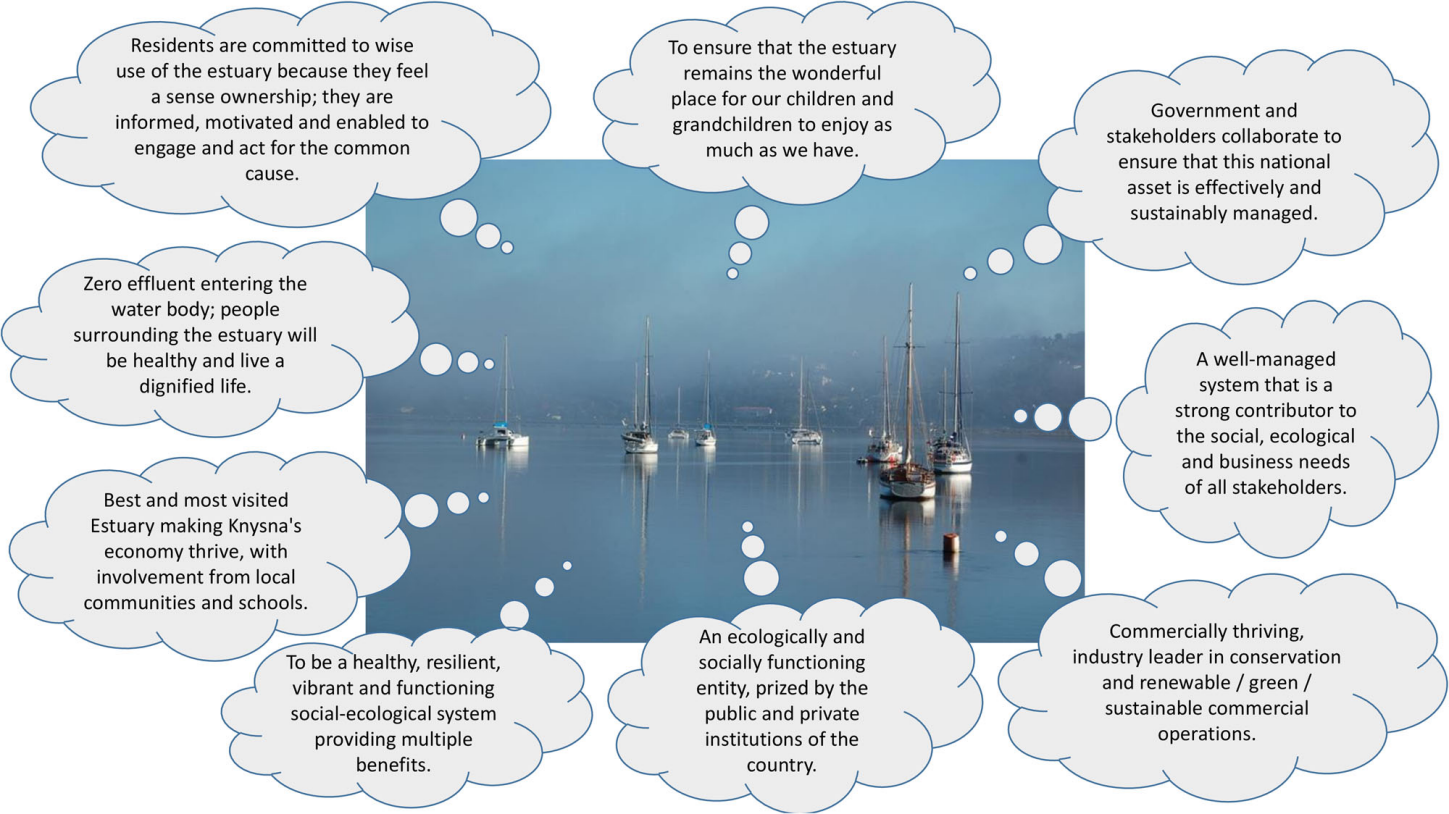
A sample of the diverse dreams that stakeholders have for the Knysna Estuary, as articulated in response to the question: What is your dream for the Knysna Estuary in the next 10 years?

Stakeholders listed more than 50 organizations, entities and interest groups to be consulted when making decisions regarding Knysna Estuary, including a range of government departments, NGOs, user groups and residents across a socio-economic gradient. Nevertheless, SANParks (79%) and Knysna Municipality (36%) were identified as primarily responsible for making the decisions. There was less clarity in the responses on who else supports or should be supporting these two agencies and how. The majority of stakeholders (85%) are aware of conflict situations regarding the management of Knysna Estuary, and many have been impacted by such conflicts (30%). Most stakeholders (78%) believe that there are at least some mechanisms in place to resolve conflict, but these are not necessarily sufficient or fully effective. Respondents suggested that decision-makers and stakeholders either mostly (19%) or partly (56%) work together, while 19% thought there were very limited collaborative approaches. Most stakeholders (58%) actively participate in one or more forums linked to the management, use or a specific interest around the Estuary, while 12% historically participated and 27% have never participated. Most stakeholders (>80%) indicated that there is at least fair agreement on a future desired state for the estuary. However, stakeholders articulated highly diverse, although not mutually exclusive, dreams or visions for the estuary (Fig. 3).

Approximately half of the stakeholders who completed the questionnaire suggested that, besides a few knowledge gaps, we already know enough about the Knysna Estuary to manage it efficiently and in a predictable manner. The other half believed that, although we have some understanding of various social and ecological aspects of the estuary, these are inter-connected, making it hard to fully predict how the ecology and people’s use of and impact on the estuary will change in future. The latter group accept the need to learn and adapt as management of the system proceeds.

### Dialogue

**Fig. 4:**
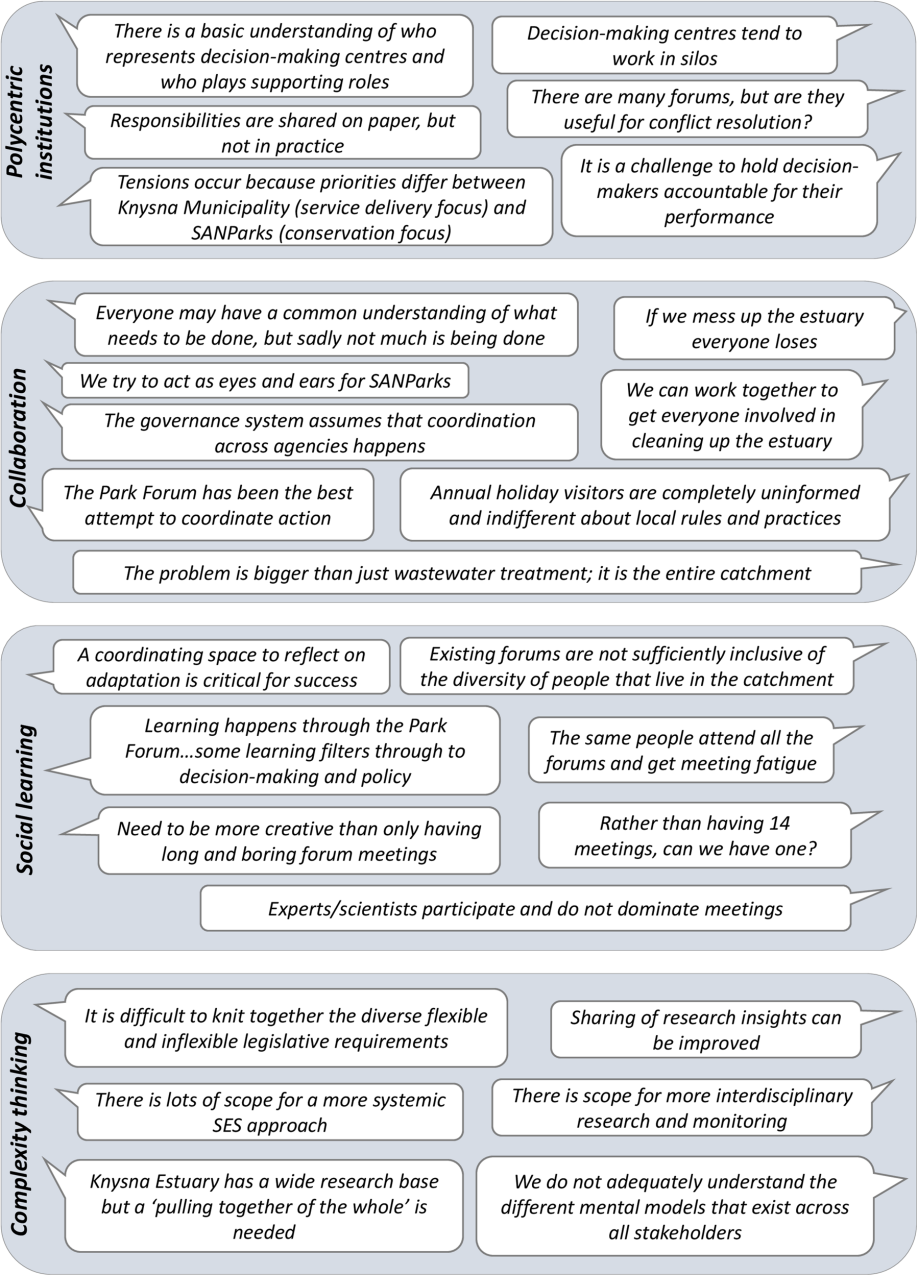
Quotes from stakeholders illustrating views that emerged from the dialogue in relation to the four selected principles of adaptive governance

Twenty-two stakeholders participated in the dialogue during June 2021, representing 15 of the 21 identified stakeholder groups (Table 1). Represented groups spanned those with direct management mandates (e.g. two provincial government departments, a district municipality and a local municipality) and those with supporting roles (e.g. user groups, interest groups, non-governmental organizations and a research entity). The latter included groups that might be perceived as with power to influence (e.g. Knysna Yacht Club and Greater Knysna Business Chamber) and those who lacked power (e.g. the fisher people) (see Table 1). Groups who did not participate were also from across the stakeholder spectrum, including the Breede-Gouritz Catchment Management Agency (local government), commercial water-based businesses and local businesses (interest groups with power), and subsidence fishers (interest groups lacking power). Figure 4 presents key quotes and insights that emerged from the dialogue.

Regarding polycentric institutions, stakeholders strongly identified SANParks and Knysna Municipality as the main managers of Knysna Estuary, confirming the findings from the pre-dialogue questionnaire. There was a perception that these two agencies have conflicting interests (conservation versus municipal service delivery), which result in a lack of progress (i.e. pulling in opposite directions) while conflict is not easily resolved because underlying tensions remain. There was almost no mention of the many other role players that were identified during the pre-dialogue questionnaire. Stakeholders frequently referred to water pollution associated with wastewater treatment and effluent discharge into the estuary as a key concern and mentioned budget and capacity constraints as obstacles to executing priority actions and law enforcement.

Regarding collaboration, stakeholders were keenly willing to collaborate but were not sure how to do so. There was a perceived lack of clarity around management responsibilities and fragmentation of approaches, resulting in a perceived lack of accountability and ownership. In addition, they felt that decision-makers only partly work together to find solutions, and issues affecting the estuary are not widely communicated with or understood by all stakeholders. Understanding is particularly poor among visiting holidaymakers, who are frequently unaware of the local ecology or the rules of use. Participants felt that much more could be done to engage people and coordinate actions by different agencies towards a common purpose. Collaborative actions have mainly focused on the major issue of effluent-related pollution and there is a clear need to broaden collaboration on other issues, engage with stakeholders across the entire catchment, and share and coordinate roles and responsibilities. The group identified that there was insufficient leadership to effectively coordinate roles and responsibilities among stakeholders, although the Park Forum offers some promise. The Park Forum, an initiative by SANParks, which aims to provide a broad platform for discussing a wide range of issues related to the Garden Route National Park, was also identified in the pre-dialogue questionnaire as a particularly important social learning space, mentioned by nine stakeholders compared to all the other forums which were only listed by one stakeholder each, possibly reflecting the broader focus of and buy-in of this forum.

Deliberations on social learning acknowledged that there are several forums for facilitating dialogue among stakeholders. However, participants felt that these forums do not sufficiently represent all stakeholders. Further, records of past discussions and decisions are not readily available to learn from, and the diffusion of information from forum discussions to the broader community is inadequate. Also, forums were perceived as largely enforcement and compliance focused rather than conducive to learning, encouragement and facilitation. Stakeholders acknowledged that forums have improved learning on water quality issues, and increased awareness of management plans and approaches. They have also enabled some information from the public to filter through to decision makers to influence policies and management decisions.

Dialogue participants who deliberated on complexity thinking suggested that various pieces of legislation do make provision for revision and adaptation of management plans in response to changing contexts. However, coordination across mandates and responsible authorities (which are often themselves rigid) is challenging and limits adaptability. Further, the general perception was that mental models that underlie understanding of the estuary among stakeholders have generally not been articulated and shared. While acknowledging that the Knysna Estuary has a wide research basis, participants suggested that both synthesis and sharing of research findings need to be improved in order to better inform policy and decision-making. Respondents also suggested that the primarily ecological focus of research on the Knysna Estuary should be complemented with transdisciplinary research that focuses on social-ecological issues and incorporates local knowledge and historically marginalized perspectives.

### Post-Dialogue Questionnaire and Appropriateness/Performance of Indicators

**Fig. 5:**
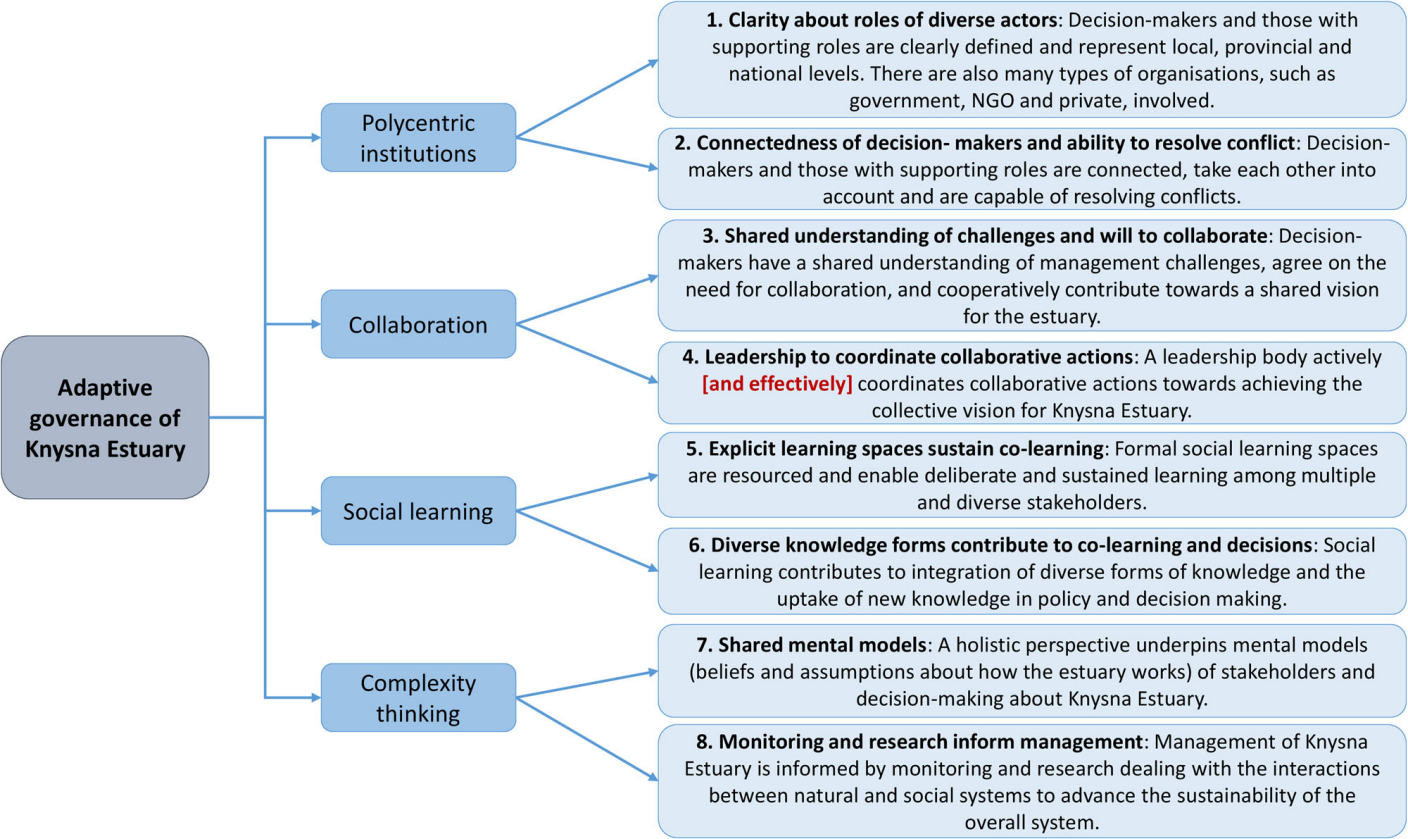
Eight indicators of adaptive governance were proposed based on the outcome of the stakeholder dialogue. For Indicator 4, the words in square brackets were added based on a suggestion made by a stakeholder in response to the post-dialogue questionnaire. This was the only suggested addition / change to the draft indicators shared with stakeholders

Of 35 invitees, 12 responded to the post-dialogue questionnaire (34% response rate) by rating the appropriateness as well as the current performance of each of the eight proposed indicators (Fig. 5).

These respondents strongly agreed that the proposed indicators reflected the issues they had discussed in the dialogue, with >90% indicating that six of the eight indicators were either appropriate or very appropriate. The lowest approval rating was for Indicator 5 at 75%, with two respondents being unsure and one indicating that the indicator was inappropriate. The latter may be explained by an optional comment that a respondent made in relation to the appropriateness of Indicator 5: “governance is setting the rules not teaching the rules”. Indicator 8 was the other indicator with a <90% approval rate, where two respondents (16.7%) selected “unsure”.

Respondents’ rating of the eight indicators in terms of current performance (Table 2) was varied: Indicators 1, 2 and 7 received relatively poor performance ratings; the performance of Indicators 3, 4 and 5 were rated as intermediate; and rating of Indicators 6 and 8 reflected the best performance. Some respondents left optional comments that could help to inform further improvement of the respective indicators (Table 2).

**Table 2:** Sample comments of respondents in relation to the current performance of the respective indicators (see Fig. 5) of adaptive governance, with the faces of the emojis representing a relative scale of 1–3, respectively corresponding to ‘good’, ‘fair’ and ‘poor’

Indicator	Respondent comments	Relative performance rating
1 Clarity about roles of diverse actors	-Good clarity for local authorities but less so for provincial and national -Primary governmental agents are clearly defined but those providing the supporting roles are not -Perhaps general public need to be better advised as to who the main role players are	
2 Connectedness of decision- makers and ability to resolve conflict	-Perhaps there is some behind the scenes interaction between the decision makers but if so it is totally invisible, e.g. no evidence of interaction at the Park Forum -Meeting regularly does not mean that conflict is resolved, that all stakeholders discuss or disclose their intentions, and more importantly that then take into consideration others intentions or mandates -Strong competing interests among different groups continues to drive conflict -I believe they do meet regularly, but not certain about how conflicts are resolved -It is not clear to me how decision-makers and those with supporting roles interact -It’s not always clear on whose land you are and different role players will give different answers on where you can or cannot fish or collect bait	
3 Shared understanding of challenges and will to collaborate	-I see absolutely no evidence of collaboration of any kind by decision makers -There is generally a shared understanding of key management challenges…Aligning actions in another thing. Priorities between structures still compete with each other -Shared understanding of management challenges do not result in action, e.g. lack of pollution control -Stakeholders generally understand the problems and need for rules but individuals may still disobey them	
4 Leadership to coordinate collaborative actions	-SANParks is a respected body and probably the only body capable of “running the show” -SANParks in Knysna is beginning to demonstrate the leadership capability. Now take that forward and play a more visible and stronger leadership role -There are certainly some leaders emerging but not enough from all organizations represented -There is a vision for Garden Route National Park but is there a specific vision for the estuary that has been collaboratively and inclusively derived? If there is, how many times is this vision explicitly considered during decision making processes? -I am not clear on who provides the leadership role for governance of the estuary	
5 Explicit learning spaces sustain co-learning	-While there are resources for occasional peer learning, there are not sufficient resources for deliberate sustained learning -Not all potential stakeholders get involved -More repeat meetings are necessary to develop a “thread of interest” -Existing spaces generally take the form of a formal presentation followed by discussion and questions. There is scope for alternative learning opportunities. -My only real interaction is with SANParks and not with other stakeholders. I would be interested to participate and engage in such social learning spaces	
6 Diverse knowledge forms contribute to co-learning and decisions	-Need to motivate non-participatory parties to get involved -I am not sure how many different forms of knowledge are integrated and included in decision making….let alone policy -Limited opportunity for engagement with other stakeholders, hence not much social learning for change -We see researchers working here and are curious. Would be interested to get feedback on what they are doing and finding	
7 Shared mental models	-Not enough diverse groups involved -Not aware of a holistic perspective -This is an area for vast improvement -People think mostly about their own interest	
8 Monitoring and research inform management	-The research being carried out is wonderful and essential but much, much more is needed -Lots of research has and continues to be conducted but most of this is discipline based -Needs to be more cooperation between researchers	

As part of the post-dialogue questionnaire, 25% of respondents indicated that they felt excited about the overall process, 50% were happy and 25% were concerned. Most respondents (83.3%) indicated that they would like to remain part of an ongoing process, while one person indicated that he/she had invested enough time in the process for now and another indicated that he/she had not been involved from the beginning and that their input would not add value at this point.

## Discussion

### Reflecting on the Process and Framework

Our research process was informed by the assumption that facilitated dialogue has the potential to, through collective interrogation of the patterns of thinking and acting, reveal new insights and possibilities, which may help to transform the understanding and behaviour of participants (Isaacs 1993). This is akin to the use of narrative strategy, also based on dialogue, where a level of coordinated action can emerge from an ever-evolving collective understanding (Nonaka and Hirose 2015) – as opposed to being driven solely by a planning process, strategy or rational action plan. Dialogue for sustained change in behaviour, as with narrative strategy, assumes a never-ending process. There are, however, early signals that our study intervention has made a positive start to such a journey. For example, although it took time and effort to motivate and enable the participation of fisher folk (Table 1) in the pre-dialogue questionnaire (four participants) and dialogue (two participants), enthusiasm for participation is growing, with 18 members of this group having participated in a more recent focus group meeting on the management of the Knysna Estuary (meeting held on September 8, 2022; attended by authors DR and MT). During this latter meeting, these stakeholders articulated a shift in their perception about their role regarding management of the estuary, from previously having to only listen to what would be done, to now being able to share their knowledge and expectations. A strong message that came from this group is that they previously felt alienated and had lost interest in conservation, but now feel motivated to clean up the estuary and educate others to do the same.

With our principle-based framework (Fig. 2; Supplement 1) we followed the suggestion that a reflexive framework could be used to both assess and catalyze learning and governance of a particular social-ecological system (Bennett and Satterfield 2018). Our framework served to focus and structure the stakeholder dialogue, and in so doing provided a theoretical basis from which to surface key issues and needs to form the basis of the eight governance indicators. The four principles selected for this study have been recognized as core factors, processes or “prescriptions” (Huitema et al. 2009) for enabling the emergence and institutionalization of adaptive governance (Chaffin and Gunderson 2016). Based on our observations during the facilitated dialogue, stakeholders found it easy to relate to these principles, and the criteria and questions listed for each principle stimulated extensive discussions. From these discussions emerged our eight indicators of adaptive governance. Therefore, although these indicators were stimulated by theory, they were articulated and empirically validated as generally regarded important features of at least Knysna Estuary’s governance.

Our principles are not mutually exclusive, for example, systems thinking (part of Principle 4 – complexity thinking) can be enabled when diverse participants engage in social learning (Principle 3), bringing together knowledge of different features of the social-ecological system (Gerlak et al. 2019). Furthermore, polycentric systems have been variously defined in the literature, potentially leading to confusion. For the dialogue, we included questions related to the sharing of responsibilities and power across a nested network of organizational units (Ruane 2020), and decentralization of decision-making (Djalante et al. 2011). However, these attributes were not part of the original conceptualization of a polycentric system, which rather emphasized a diversity of formally independent units choosing to act in ways that take account of others and are able to resolve conflict (Carlisle and Gruby 2019; Ostrom et al. 1961). The indicators for polycentric institutions that emerged from the dialogue are nevertheless strongly aligned with the concept’s authentic definition. We also note that, as pointed out by Carlisle and Gruby (2019), there is a potential tension between having the autonomous organizations of a polycentric system (Principle 1) and the convergent perspectives and shared theory of change required for collaboration (Principle 2). In this regard, the stakeholders in our case study suggested that SANParks and Knysna Municipality mostly operate independently, that they are perceived to have conflicting mandates and are lacking in collaboration. Steps towards improving the performance of the indicators identified in Table 2 (in particular indicators 1–5), would likely improve this situation.

### Indicators as Dynamic Narratives of Change

We propose that the eight narrative indicators that emerged from our stakeholder dialogue represent a collective ‘theory of change’ for achieving a desired state of governance to better support the effective management of the Knysna Estuary. Moreover, the indicators provide a basis for an emergent leadership group (potentially led by SANParks) to facilitate ongoing dialogue and promote reflexivity among stakeholders. Reflexivity is a key virtue providing responsiveness in environmental governance, defined as “the ability of a structure, process, or set of ideas to reconfigure itself in response to reflection on its performance” (Dryzek and Pickering 2017; page 353). This is particularly important in adaptive governance, which responds to “the societal need and desire to adapt to changing conditions” (Chaffin et al. 2016; page 404). In the Knysna estuary context, reflexivity would also imply that stakeholders remain open to modification and possible expansion of the indicators as and when the evolving collective thinking deems such updates necessary.

We further see potential for regular (e.g. annual) co-reflection on the indicators and their perceived performance in strengthening the institutionalization of adaptive governance of the Knysna Estuary. Participatory co-reflection of this nature would help to transcend some of the struggles that adaptive governance has had in practice elsewhere in the world (Chaffin et al 2016; Chaffin and Gunderson 2016). The evaluation of progress towards the co-designed indicators in a participatory setting provides practical steps for bridging between adaptive governance as a concept and adaptive governance in practice. In our study context, practical steps to address current shortcomings identified by stakeholders (Table 2) can contribute to such bridging. For example, this could include prioritizing regular meetings between relevant stakeholders as part of annual plans and budgets (Indicators 1 and 2); a formal agreement between SANParks and Knysna Municipality to collaborate on aspects of the estuary’s management (Indicator 3); and allocating realistic budgets for supporting social learning fora (Indicator 5) and monitoring of key social-ecological feedbacks (Indicator 8).

Furthermore, in a dynamic social environment where existing stakeholders leave and new stakeholders enter the system, ongoing dialogue is essential for ensuring continuity and renewal of a collective understanding of governance principles and processes among stakeholders. We mentioned our promising observation of how the participation of the fisher people increased in numbers and confidence during the course of the dialogue and beyond. Ideally, similar diffusion of new understanding and buy-in would be required from formal organizations in the polycentric system. This may be more challenging in organizations such as the Knysna Municipality where an individual from one department (e.g. environmental management) may not have much influence in another department (e.g. waste management). Also, specific attention may have to be given to identifying and correcting misunderstandings, e.g. confusing social learning with training as demonstrated in a stakeholder response to the second questionnaire.

### Complementarity Between Adaptive Management and Adaptive Governance

In the introduction we suggested that the adaptive management of complex systems can be facilitated by adaptive governance arrangements. Although SANParks’ experience with applying Strategic Adaptive Management spans more than two decades, to date the agency has given relatively little practical consideration to adaptive governance. To promote the incorporation of adaptive governance as part of SANParks’ current management approach, it would make sense to conceptually show the complementarity between Strategic Adaptive Management and adaptive governance.

**Fig. 6:**
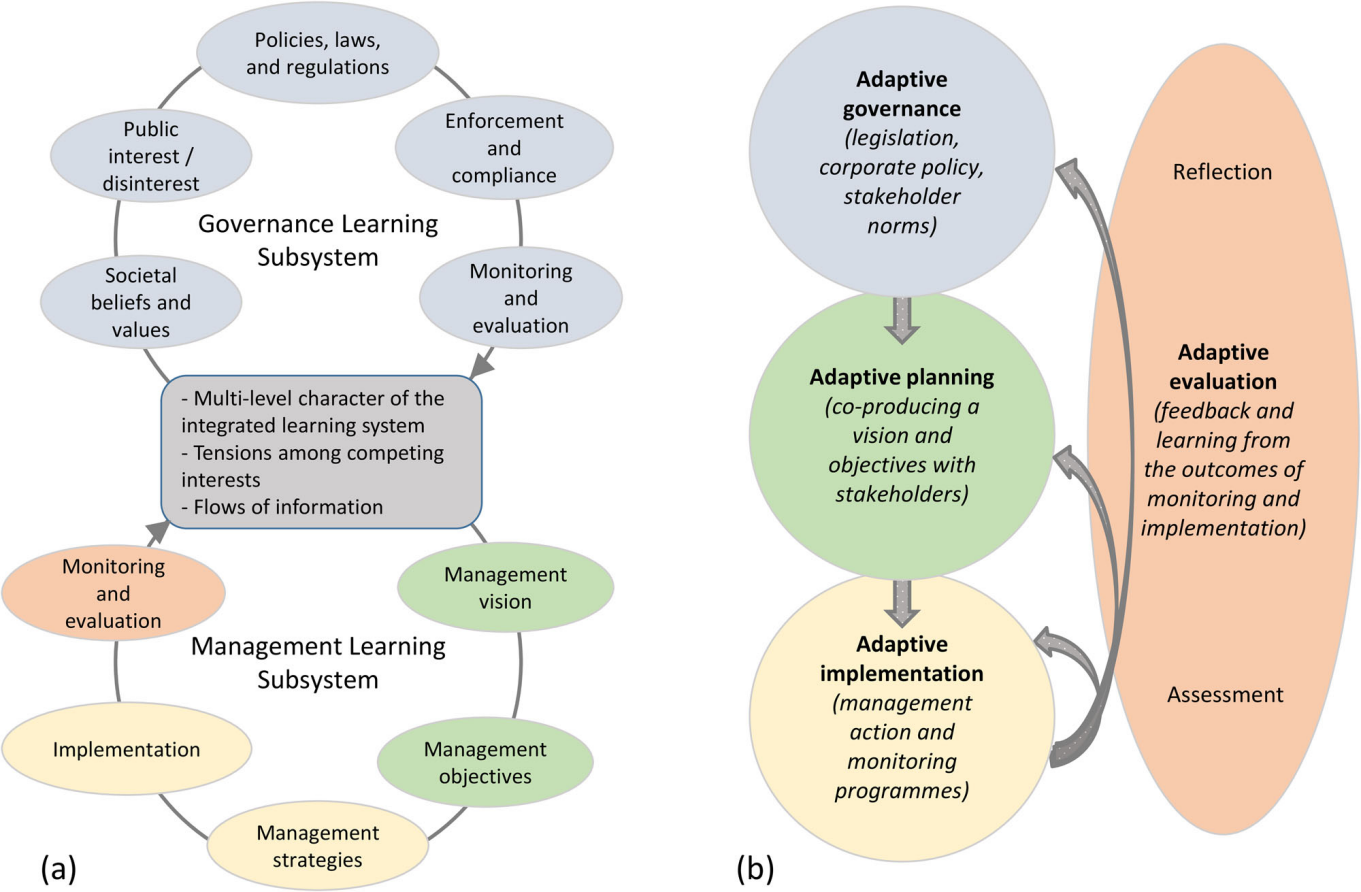
Different conceptualizations of the relationship between adaptive management and adaptive governance: (**a**) as two sub-systems of an integrated learning system (modified from Nkhata and Breen 2010) and (**b**) as a subsystem of Strategic Adaptive Management (modified from Novellie et al. 2016). In (**b**), downward arrows indicate sequential dependencies and curved arrows learning feedback. Color-coding indicates corresponding steps/sub-processes across the respective conceptualizations

In the context of public protected areas, Nkhata and Breen (2010) propose that adaptive management and adaptive governance can be viewed as two sub-systems of an integrated learning system (Fig. 6a). Accordingly, governance provides direction and a means of resolving trade-offs in order to establish the public interest, while management offers the means of transforming the public interest into reality. In turn, the practical lessons generated by on-the-ground management actions and experimentation feedback to influence the formation of public interest in the governance cycle (Nkhata and Breen 2010). Thus, in South Africa’s current national park context, “while society expresses its demands at a higher level through governance processes in such a way as to construct the public interest in a protected area, protected area agencies are expected to successfully accomplish the public interest at a lower level through management processes” (Nkhata and Breen 2010; page 405).

**Fig. 7:**
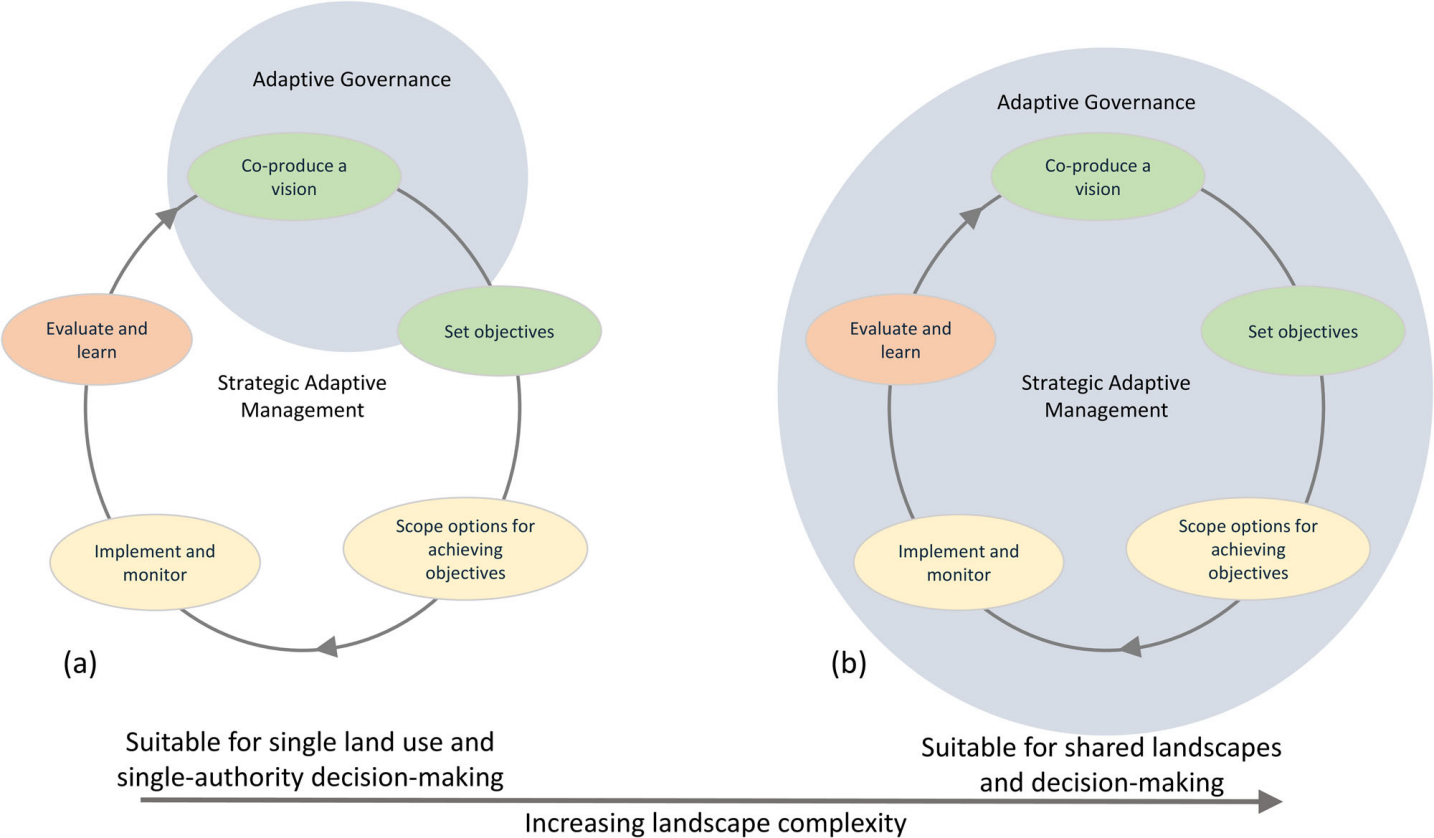
An increased emphasis on adaptive governance is required with an increase in landscape and decision-making complexity, from relatively simple contexts where governance arrangements mainly direct visioning and objective setting (**a**) to complex social-ecological settings where diverse stakeholders actively engage in processes of dialogue, social learning and collaboration to enrich every step of the adaptive management cycle (**b**)

More recently, adaptive governance has been conceptualized as a sub-process of SANParks’ Strategic Adaptive Management (Fig. 6b; Novellie et al. 2016), referring to the evolving ‘rules of the game’ at a range of levels, from national legislation to park policy and local rules shaped by stakeholder norms, values and relationship. These rules provide the context within which a vision and objectives are co-produced with stakeholders (adaptive planning; Fig. 6b). This depiction seems appropriate for relatively simple contexts such as fenced parks with single-authority implementation of the co-produced vision and objectives. However, for our case study of the Knysna Estuary, we see adaptive governance as more encompassing than only feeding into visioning and objective setting; rather it provides a social context that plays an enabling role for every step of the adaptive management cycle (Fig. 7).

We propose that ongoing co-reflection on the eight indicators of adaptive governance (as part of ongoing dialogue among stakeholders) will help to catalyze adaptive governance by, for example: clarifying the various role players in the polycentric system and improving the likelihood of effective conflict resolution (Principle 1); promoting a shared understanding of problems and emergent leadership to strengthen collaboration (Principle 2); facilitating social learning and acknowledgement of diverse knowledge forms in decision making (Principle 3); and generating a more holistic perspective of the estuary as a coupled social-ecological system and related information from monitoring and research (Principle 4). At the same time, pursuing the conditions encapsulated by the indicators of adaptive governance will enable adaptive management for complex settings in several ways. First, ongoing social learning (Principle 3) will help to maintain the relationships and trust that are typically created during visioning and objective setting but tend to weaken during subsequent steps when SANParks largely takes sole responsibility for implementation (Roux et al. 2021). Second, formalizing collaborative agreements (Principle 2) between key organizations, e.g. SANParks and Knysna Municipality in our case study, will necessitate agreement on shared concerns and joint setting of objectives as well as performance indicators, and also joint scoping of options through which to achieve these. Accountability can be broadened further by involving stakeholders in supporting roles in these steps (Principle 1), who during the dialogue indicated their keenness to support SANParks. Third, implementation can be enriched through collaborative decision-making informed by a systems understanding of, and inter- and transdisciplinary research on, the estuary (Principle 4).

## Conclusion

Our study represents place-based research where local context matters. We explore how a conservation agency (SANParks) can make its standard adaptive management approach work in settings where it has legitimacy to manage but lacks overarching authority over other organizations (with overlapping mandates), resource users and stakeholders. Our research was guided by suggestions in the literature that (a) adaptive governance can enable adaptive management in such complex contexts, and (b) governance indicators that are developed in collaboration with stakeholders can help to facilitate collective reflection, learning and adaptation towards desirable governance conditions.

With the open-access Knysna Estuary as a case study, we designed an engaged research approach centred on a facilitated stakeholder dialogue. The dialogue formed the basis for co-reflecting on the state of governance of the estuary and co-producing a set of eight indicators that might help to guide a transition to improved governance. However, such a transition will be dependent on ongoing leadership from SANParks (or another mandated agency) to maintain a relatively inclusive stakeholder dialogue through which the indicators can be used as reflexive monitoring and evaluation tools.

At the local scale, our research represents an exploratory step for SANParks to incorporate adaptive governance as part of its Strategic Adaptive Management, specifically in contexts that necessitate a deeper level of stakeholder engagement and collaboration. More generally, our case study from the Global South presents an approach – including the principle-based framework, facilitated dialogue and process for crafting indicators – that could serve as a departure point for research interventions in similar contexts elsewhere. Furthermore, we add to the largely conceptual literature on the interplay between adaptive management and adaptive governance by contributing an example of how this interplay might work in the real-world context of a conservation agency (SANParks).

### Supplementary Information

Supplement 1

Supplement 2

Supplement 3
