# Supplementary material for: Co-Producing Narratives and Indicators as Catalysts for Adaptive Governance of a Common-Pool Resource within a Protected Area
Source: Environ Manage. 2023 Sep 23;72(6):1111–27. doi: 10.1007/s00267-023-01884-z (PMC10570219; doi:10.1007/s00267-023-01884-z)
Supplement: Supplementary file 1 — Supplement 1 [file 267_2023_1884_MOESM1_ESM.pdf]

Supplement 1

Principle-based framework for facilitated dialogue on adaptive governance of the Knysna Estuary

Introduction

Our framework consists of four common principles of adaptive governance (e.g. Ruane 2020; Huitema et al. 2009; Novellie et al. 2016) and a number of criteria and associated questions for each principle, as derived from the literature.

Principle 1: Polycentric institutions

Polycentric systems have been variously defined in the literature, potentially leading to confusion. Carlisle and Gruby (2019) adopt the set of attributes of a polycentric system identified by Vincent Ostrom et al. (1961) in a seminal paper: (1) units of the system are formally independent of one another; (2) they choose to act in ways that take account of others, (3) through processes of cooperation, competition and conflict resolution. Carlisle and Gruby (2019), while noting the existence of various definitions in the literature, argue in favour of loyalty to the original concept, rather than imagining substantively different concepts. They point out that polycentricity has sometimes been used interchangeably with organization in multiple layers of nested enterprises. In their view, polycentricity is more than a nested governance system where decision-making authority is distributed across diverse institutional scales and between a multiple network of actors (e.g. as defined recently by Ruane, 2020). Although systems may be characterized as being polycentric, it does not necessarily mean that the arrangement actually functions as a polycentric system. A polycentric governance system exists "if the decision-making centres take each other into account in competitive and cooperative relationships and are capable of resolving conflicts" (Carlisle and Gruby 2019: p. 928).

In the definition of Carlisle and Gruby (2019) responsibilities and power are not necessarily shared between different decision-making centres, nor is decision-making power necessarily decentralised to local bodies. Instead the decision-making centres are envisaged as being linked, and taking one another into account, despite being in competition - a state described as "coopetition" (Bengtsson & Kock 2014, cited in Heslinga et al. 2019).

In choosing the criteria for polycentricity listed below we considered all the above interpretations of polycentricity, which gave participants in our stakeholder dialogue an opportunity to reflect on the range of interpretations in the literature. We note that the first two questions under 1.1 below do not accord with the original definition of polycentricity as clarified by Carlisle and Gruby (2019), but rather with nesting in multiple layers. All the remaining questions in criteria 1.1 and 1.2 are consistent with the original definition.

Criteria for achieving the principle of polycentric institutions:

1.1 Responsibilities are shared and decision-making authority passed to the lowest level possible

- Are responsibilities shared across scales (national, provincial and local) and between public and private role players?

- Is decision-making authority and management responsibility for the Knysna Estuary passed to the lowest level possible?
- Where responsibilities are distributed across the multi-level system, is it still possible to hold decision makers accountable for their performance?
- Does the Knysna Estuary, in your opinion, operate as a multi-level system where different decision-makers operate, though are mindful of one another's roles and responsibilities, and cooperate or where necessary work jointly, to solve problems and resolve issues?

1.2 Polycentric institutions is supported by appropriate knowledge and mechanisms for informal and formal engagement

- Is there a clear understanding of who represents decision-making centres (any independent group making norms and rules – even if they are unwritten rules – within a specific domain) and who play critical supporting roles (those who lack authority to make decisions but exert influence on policies or provide critical technical or financial support)?
- Are the different decision-making centres (who work largely independently) taking each other into account in competitive and cooperative relationships, and are they capable of resolving conflicts?
- Are there low-cost mechanisms for conflict resolution in place?
- Does the nature of competition and conflict support self-organisation, learning and change, i.e. it is not so intense that it undermines effectiveness and ability to adapt?
- Are the transaction costs (i.e., costs of consultation, reaching agreements and enforcing agreements) of ensuring coordination across the multi-level system provided for?

Principle 2: Collaboration

Like polycentric governance, collaborative governance has been inconsistently defined (Emerson et al. 2012). Ansell and Gash (2008, 544) define collaborative governance as: ''*A governing arrangement where one or more public agencies directly engage non-state stakeholders in a collective decision-making process that is formal, consensus-oriented, and deliberative and that aims to make or implement public policy or manage public programs or assets.*''

Emerson et al. (2012, p 2) propose a broader definition than that of Ansell and Gash, namely: "*the processes and structures of public policy decision making and management that engage people constructively across the boundaries of public agencies, levels of government, and/or the public, private, and civic spheres in order to carry out a public purpose that could not otherwise be accomplished*".

A key feature of collaborative governance is a shared theory of change which is built on a shared understanding of the source of the problem and the goals of the landscape (Emerson and Nabatchi 2015, cited in Rapp 2020). Trust, both interpersonal and inter-organisational, is a key driver of learning and shared motivation (Emerson et al. 2012, Rapp 2020). Following from the definition of collaborative governance of Emerson et al. (2012), a transition from divergent perspectives to convergent perspectives and greater collaboration may hold value if:

1. The decision-making centres are interdependent, they share a common purpose that could not be achieved by any of them acting independently.
2. There is collective uncertainty as to how the SES should be managed. Uncertainty, the primary challenge in managing 'wicked' systems, is a driver of collaboration (Emerson et al. 2012). If

the parties had perfect information they would be able to act independently to pursue their own interests.

3. There are consequential incentives for collaborative action (Emerson et al 2012). Such incentives may be negative, for example inattention to a common threat may have negative consequences for all. Or they may be positive, for example a grant or new funding opportunity could lead to the development of a collaborative initiative.
4. Levels of trust (dispositional, rational, affinitive and procedural – Rapp 2020) are sufficient to drive learning and shared motivation to collaborate.
5. There is leadership capacity, a coordinating body or unit can promote coordination of roles, functions and mandates of different governments, agencies and stakeholder organizations/groups.

In summary: The principle of collaboration acknowledges that many challenges associated with complex systems such as Knysna Estuary are too big for one organisation to address alone. Collaborative governance is the combination of structures and processes which engage people (across agencies, levels of government, and private, civic and public spheres) to achieve a shared purpose like maintaining the health of the estuary (Emerson et al. 2012). Requirements for collaborative governance are development of a shared understanding of the nature or source of the problem and the goals for the broader environment (or social-ecological system), trust, and capable leadership. A combination of a variety of autonomous organisations (polycentric governance), with some working together to solve a common problem (collaborative governance) provides the most scope for effective common pool resource governance.

Criteria for achieving the principle of collaboration:

2.1 There is a shared understanding of the problem(s) and the goals of the landscape/waterscape

- Is there collective understanding of the management problems of Knysna Estuary?
- Is there collective uncertainty among stakeholders as to how to manage problems, and is this uncertainty a motivation to collaborate?
- Has a common purpose or vision for the future of Knysna Estuary been described, and if so do you think most persons or organisations understand and agree with the purpose or vision?
- If there is a need for change in the Knysna Estuary system, is there a common understanding of how such change can be achieved?

2.2 Diverse actors work in conjunction and share resources for a common purpose

- Are the decision-making centres in the Knysna Estuary dependent on each other in that they are unable to accomplish the joint public purpose by acting on their own?
- Are the decision-making centres engaging people constructively to achieve a purpose that could not otherwise be accomplished?
- Is there one leadership body or unit that promotes coordination of the roles, functions and mandates of different agencies and stakeholders?
- Are there incentives (either positive such as gaining something, or negative such as avoiding a loss) for collaborative action?
- Is collaboration characterized by consensus-oriented decision making and pooling of resources?

Principle 3: Social learning

Social learning is a governance instrument which has potential to build resilience in social-ecological systems (De Kraker 2017). Reed et al. (2010) define social learning as "a change in understanding that goes beyond the individual to become situated within wider social units or communities of practice through social interactions between actors within social networks". Social learning emphasizes deliberate and sustained interaction between multiple stakeholders, who may have diverse and even diverging views about resource management objectives and preferred actions, with an increase in trust, convergence of perspectives, and improved decision making being key outcomes (Cundill and Rodela 2012; Van der Wal et al. 2014).

De Kraker (2017) cautions that there are not many situations where social learning can be fostered as a governance instrument, or where there is a good chance of achieving the desired outcomes. He identifies the following case-related conditions that promote social learning.

1. There is a balance of power and strong interdependence between stakeholders in solving the problem.
2. There are overlapping values and interests, trust, and limited risk.
3. The institutional context is supportive, and there is a sense of urgency and awareness of the need for change. (Crises in the system may induce the required sense of urgency.)
4. There is effective leadership or facilitation, space for reflection, a safe informal environment, and transparency.
5. Resources are available for long-term engagement and repeated meetings.
6. Conceptualisation and operationalisation of social learning are explicitly defined.
7. There are good connections between informal social learning processes and formal policy processes.
8. Learning spaces can be opened up to a diversity of stakeholders, and are not expert-dominated.

Criteria for achieving the principle of social learning:

3.1 Deliberate and sustained interaction among multiple and diverse stakeholders (who may have diverse and even differing views about resource management objectives and preferred actions) is enabled

- Are there official forums/spaces for collective deliberation and reflection?
- Are opportunities for discussion and learning at these forums open to all stakeholders?
- Are both public and private stakeholders participating in these forums in a transparent and trusting manner, and the forums not dominated by experts?
- Is there effective leadership or facilitation of meetings that enable meaningful interaction?
- Are resources available for long-term engagement and repeated meetings?

3.2 Deliberation among stakeholders leads to an increase in trust, convergence of perspectives, and improved decision making

- Is deliberation leading to new understanding and behaviour among stakeholders?
- Is informal social learning contributing directly to formal policy determination and decision making?

Principle 4: Complexity thinking

Social-ecological systems need to be understood as complex adaptive systems (CAS). Outcomes in such systems are uncertain and poorly predictable and governance systems that assume predictability are likely to go wrong (Preiser et al. 2018; Biggs et al., 2012; Cilliers et al., 2013; Craig, 2010; McCool et al., 2015; Snowden and Boone, 2007). An example of this, relevant to the Knysna Estuary, is the National Environmental Management: Protected Areas Act, which assumes that the management needs of protected areas are predictable over time, and hence that management plans can be drawn up and left to roll out without frequent revision and adaptation (Novellie et al. 2016). Models are essential tools for dealing with complex systems (Cilliers et al. 2013). It is particularly important that the mental models that underpin the management of complex social-ecological systems (for example the common theories of change of Emerson et al. 2012) reflect complexity thinking and do not make unwarranted assumptions of predictability. CAS are not the domain of individual expertise, but require broadened and diversified participation at multiple interconnected levels (Snowdon and Boone 2007; Cilliers et al. 2013). Information on the state of the system, and its users, is critical to the management of CAS (Dietz et al. 2003). Openness to new ideas, sharing of information, experimentation, learning and monitoring are essential to managing in the face of unpredictable change.

Preiser et al. (2018) propose a conceptual typology of six organizing principles of CAS based on a comparison of leading scholars' classifications of CAS features and properties. They aim to provide an entry point to researchers, decision makers, who want to operationalize CAS-based approaches in the study of social-ecological systems. The strategies for studying and engaging with CAS identified by Preiser et al. (2018) are: i) develop an integrated understanding of social-ecological systems by exploring a variety of models that span a broad spectrum of methodologies and disciplinary divides; ii) move from studying the characteristics of individual parts in isolation to look at systemic properties that emerge from the underlying pattern of organisation; iii) understand underlying organisational processes, connections and emergent behavioural patterns; iv) recognise that the system is open, not closed, there is no inside and outside; v) assess relations, connections, and multiple complex causal pathways; and vi) characterise networks and cycles so as to facilitate our ability to anticipate adaptive and transformative behaviour and pathways.

Arlinghaus et al. (2017) advocate managing freshwater recreational fisheries as complex adaptive social-ecological systems. They recommend the following: shift from disciplinary to inter- and sometimes transdisciplinary research; focus on an adaptive, flexible and enabling form of governance and management, rather than a command and control one; social-ecological feedbacks and processes should be managed; critical slow variables, that either drive the system or potentially maintain it in an undesirable state, should be monitored and managed; and social and ecological diversity should be managed and maintained.

The strategies advocated by Preiser et al. (2018) and Arlinghaus et al. (2017) can be summarised as:

- Underlying mental models, the essential common theories of change, take into account complexity thinking.
- Participation is broad and diverse.
- Innovation and experimentation is encouraged and success and failures are monitored.
- Information and learning are shared. A higher risk tolerance is embodied.
- Adaptability: Processes exist to revisit and evolve policies, institutions and adapt actions
- Flexibility: Policies exist that recognize the need to downscale environmental management and conservation models

- The SES is recognised as being open, not closed; a systemic ecosystem approach, whereby ecosystems are viewed as a whole, informs management decisions.

The ideal governance system should explore the extent to which these strategies are being applied, and promote them where necessary. An approach to examine the diversity of mental models, and perceptions on complexity thinking, is through bottom-up "reconstructive" stakeholder analysis (Reed et al. 2009).

Criteria for achieving the principle of complexity thinking:

4.1 Social-ecological systems are understood and managed as complex adaptive systems

- Is guiding legislation acknowledging the unpredictability of social-ecological systems and enabling frequent revisions and adaptations of management plans?
- Are processes in place to revisit and evolve policies and institutions, and adapt actions?
- Do underlying mental models of Knysna Estuary reflect complexity thinking?
- Are key driving variables of the system monitored and the information made available?

4.2 Diversity of perspectives and ongoing learning are embraced

- Is understanding of the Knysna Estuary also informed by systemic (i.e. considering the whole social-ecological system) and transdisciplinary (i.e. involving knowledge from several disciplines as well as from managers, scientists and resource users) research, as opposed to primarily research that focus on single components of the estuary or single research disciplines?
- Is experimentation and innovation encouraged (with a fair degree of risk tolerance), and successes and failures monitored?
- Is ongoing monitoring linked to purposeful learning, sharing of information and adaptation of plans when required?
- Is the better understanding of problems, and making of important decisions, based on broad and diverse participation by stakeholders at multiple interconnected levels?

4.3 A systemic ecosystem approach, whereby ecosystems are viewed as a whole, informs management decisions

- When making decisions, is consideration given to the broader estuary catchment including its human, economic and ecological components and their interactions?

References

Ansell C, Gash A. 2008. Collaborative governance in theory and practice. Journal of Public Administration Research and Theory 18: 543–71.

Arlinghaus R, Alós J, Beardmore B, Daedlow K, Dorow M, Fujitani M, Hühn D, Haider W, Hunt LM, Johnson BM, Johnston F, Klefoth T, Matsumura S, Monk C, Pagel T, Post JR, Rapp T, Riepe C, Ward H, Wolter C. 2017. Understanding and managing freshwater recreational fisheries as complex adaptive social-ecological systems. Reviews in Fisheries Science & Aquaculture 25(1): 1-41. https://doi.org/10.1080/23308249.2016.1209160

Biggs R, Schlüter M, Biggs D, Bohensky EL, Burnsilver S, Cundill G, Dakos V, Daw T, Evans L, Kotschy K, Leitch A, Meek C, Quinlan A, Raudsepp-Hearne C, Robards M, Schoon ML, Schultz L, West PC. 2012.

Towards principles for enhancing the resilience of ecosystem services. Annu. Rev. Environ. Resour. 37: 421–448.

Carlisle K, Gruby RL. 2019. Polycentric systems of governance: a theoretical model for the commons. Policy Studies Journal 47(4): 927-952.

Cilliers P, Biggs HC, Blignaut S, Choles AG, Hofmeyr JS, Jewitt GPW, Roux DJ. 2013. Complexity, modeling, and natural resource management. Ecology and Society 18, 1. http://dx.doi.org/10.5751/ES-05382-180301

Craig RK. 2010. Stationarity is dead: long live transformation: five principles for climate adaptation law. Harvard Environmental Law Review 34: 9–73.

Cundill G, Rodela R. 2012. A review of assertions about the processes and outcomes of social learning in natural resource management. Journal of Environmental Management 113: 7-14.

De Kraker J. 2017. Social learning for resilience in social-ecological systems. Current Opinion in Environmental Sustainability 28:100–107. http://dx.doi.org/10.1016/j.cosust.2017.09.002

Dietz T, Ostrom E, Stern PC (2003) The struggle to govern the commons. Science 302(5652):1907-1912. http://dx.doi.org/10.1126/science.1091015

Emerson K, Nabatchi T, Balogh S. 2012. An integrative framework for collaborative governance. Journal of Public Administration Research and Theory 22: 1-29.

Heslinga J, Groote P, Vanclay F. 2019. Strengthening governance processes to improve benefit-sharing from tourism in protected areas by using stakeholder analysis. Journal of Sustainable Tourism 27(6): 773-787. https://doi.org/10.1080/09669582.2017.1408635

Huitema D, Mostert E, Egas W, Moellenkamp S, Pahl-Wostl C, Yalcin R (2009) Adaptive water governance: assessing the institutional prescriptions of adaptive (co-)management from a governance perspective and defining a research agenda. Ecology and Society 14(1): 26.

McCool SF, Freimund WA, Breen C. 2015. Benefiting from complexity thinking. In: Worboys GL, Lockwood M, Kothari A, Feary S, Pulsford I (Eds). Protected Area Governance and Management. ANU Press, Canberra, pp. 291–326.

Novellie P, Biggs H, Roux D (2016) National laws and policies can enable or confound adaptive governance: examples from South African national parks. Environmental Science & Policy 66:40–46.

Ostrom E. 1990. Governing the Commons: The Evolution of Institutions for Collective Action. Cambridge University Press, Cambridge, UK.

Ostrom V, Tiebout CM, Warren R. 1961. The organization of government in metropolitan areas: a theoretical inquiry. American Political Science Review 55(4): 831-842.

Preiser R, Biggs R, De Vos A, Folke C. 2018. Social-ecological systems as complex adaptive systems: organizing principles for advancing research methods and approaches. Ecology and Society 23(4):46. https://doi.org/10.5751/ES-10558-230446

Rapp C. 2020. Hypothesis and theory: collaborative governance, natural resource management, and the trust environment. Frontiers in Communication 5:28. https://doi.org/10.3389/fcomm.2020.00028

Reed MS, Evely AC, Cundill G, Fazey I, Glass J, Laing A, Newig J, Parrish B, Prell C, Raymond C, Stringer LC.2010, What is social learning? Ecology and Society, 15:1-10.

Ruane S. 2020. Applying the principles of adaptive governance to bushfire management: a case study from the South West of Australia. Journal of Environmental Planning and Management 63: 1215-1240. https://doi.org/10.1080/09640568.2019.1648243

Snowden DJ, Boone ME. 2007. A leader's framework for decision making. Harvard Business Review 85(11): 1-8.

Van der Wal M, De Kraker J, Offermans A, Kroeze C, Kirschner PA, Van Ittersum M. 2014. Measuring social learning in participatory approaches to natural resource management. Environmental Policy and Governance 24(1): 1-15. https://doi.org/10.1002/eet.1627
