# Supplementary material for: Co-Producing Narratives and Indicators as Catalysts for Adaptive Governance of a Common-Pool Resource within a Protected Area
Source: Environ Manage. 2023 Sep 23;72(6):1111–27. doi: 10.1007/s00267-023-01884-z (PMC10570219; doi:10.1007/s00267-023-01884-z)
Supplement: Supplementary file 2 — Supplement 2 [file 267_2023_1884_MOESM2_ESM.pdf]

Knysna Estuary Stakeholder Survey

Dear stakeholder,

Knysna Estuary is a critically important ecosystem, from a biodiversity as well as a socio-economic perspective. The estuary provides multiple benefits to diverse users, and stakeholder expectations are equally diverse and sometimes seemingly incompatible with one another. Managing such systems is notoriously difficult, because there are often no obvious right or wrong answers, only trade-offs between multiple and conflicting viewpoints and management options. Appropriate governance systems are needed to consider and navigate this trade-off space.

SANParks has initiated a process to reflect on the current governance system for Knysna Estuary and identify possible shortcomings, and to co-develop with stakeholders a set of indicators that can serve as governance ideals to strive for. We have taken care to identify a group of stakeholders that are representative of i) key user groups, and ii) spheres of government active in the governance of Knysna Estuary. You are one of these stakeholders and we do hope that you will be willing to participate in this process to help co-design indicators for the governance of the estuary.

One way of contributing towards this process is by completing a short voluntary and anonymous questionnaire that will i) provide you with background on the governance principles that we will use to guide our project, and ii) provide us with valuable information on the range of perspectives that exist among stakeholders. The questionnaire will take about 20 minutes to complete, and contain some questions where you need to tick a box, as well as questions where you can provide more detail in a written response.

Please note that your answers are only saved once you click on the "submit" button on the last page of the survey. As such, ensure you have adequate time to complete the survey from front to end and that you have stable internet connectivity.

Please complete the survey by 4 June.

We do hope that you will be able to prioritise time for participation in this important process.

Thank you in advance for your time.
Best regards
(Name anonymized) and team

---

*Required

Consent to participate

Please read through the following statements before you agree to participate in the study.

I understand that:
- My participation is voluntary
- No one will know what answers I provided
- I am older than 18 years
- At the end, the information provided in this survey will be used in reports, publications and presentations. Part of the results may be published online.
- The publications, reports and presentations above will combine the information from all people that have participated in the survey. These publications will not include any information about any individual, so no one will know what I said. Specific quotes from my answers may be used, but these will be used anonymously as no identifying information is required in the survey.
- I am free to leave the survey at any time or skip any questions. My responses are submitted at the end of the survey when I select the “Submit” button. Once submitted, I will not be able to withdraw my response since the researchers will have no way of identifying my response.
- The study has been reviewed and approved by the Rhodes University Ethics Committee

For questions, comments, or complaints, please email (name and email address anonymized) or (name and email address anonymized), and we will aim to get back to you within 2 weeks.

1. Do you consent to participate in the study? *

Mark only one oval.

- ◯ Yes
- ◯ No

Governance systems

2. Who should be responsible and ultimately held accountable for decision-making in the Knysna Estuary (you can mark multiple options here)?

Tick all that apply.

- ☐ National public authority
- ☐ Provincial public authority
- ☐ Local public authority
- ☐ Private Role players
- ☐ Other: ____________________

3. Please name the specific authorities, organizations, entities or interest groups you think should be CONSULTED DURING DECISION-MAKING in the Knysna Estuary. Please list all the groups you consider relevant. Please note that this does not have to reflect the current situation, but what you would consider desirable.

4. Please name the specific authorities, organizations, entities or interest groups you think should be RESPONSIBLE FOR DECISION-MAKING in the Knysna Estuary. Please list all the groups you consider relevant. Please note that this does not have to reflect the current situation, but what you would consider desirable.

5. Are you aware of conflict between the different decision-makers and stakeholders around the governance/management of the Knysna Estuary?

Mark only one oval.

- ◯ Yes, I am aware of conflict situations around the governance/management of the Knysna Estuary, and I (or my organization) have been directly involved/impacted in some of the conflict situations *Skip to question 6*
- ◯ Yes, I am aware of conflict situations around the governance/management of the Knysna Estuary but I (or my organization) have not been directly impacted/involved *Skip to question 6*
- ◯ No, and I am not aware of any conflict situations around the governance/management of the Knysna Estuary *Skip to question 10*

Conflict resolution

6. Do you feel that there are mechanisms in place to resolve conflict experienced around the Knysna Estuary governance/management?

Mark only one oval.

- Definately
- Partly
- Definitely not
- Don't know

7. Please explain your answer above.

Affected parties

8. Which groups are most affected by decisions around the governance and management of the Knysna Estuary?

9. What mechanisms are in place or can be used to ensure that those listed above have a voice and some influence on decision-making and managing of the Knysna estuary?

Important considerations in Knysna Estuary management

10. What are the most important social and ecological aspect(s) that should be considered to inform management decisions of the Knysna Estuary?

Collaboration

11. Do you think there is in general a common vision between decision-makers and stakeholders on what the Knysna Estuary should be managed towards in future? Please note that this question does not ask about agreement in terms of the details of how the estuary should be managed, but rather the ultimate goal of how we want the estuary to look like in future, the so-called future desired state of the estuary?

Mark only one oval.

- Definitely – most decision-makers and stakeholders are in agreement on the future desired state for the estuary
- Partly – a fair number of decision-makers and stakeholders agree on the future desired state of the estuary
- Definitely not – there is very little agreement on what decision-makers and stakeholders see as the future desired state of the estuary
- Not sure
- Other: ____________________

12. In your experience do you think that people with an interest in the Knysna Estuary work together to find common solutions, or do you feel that each decision-maker and stakeholder only work towards furthering their own agenda?

Mark only one oval.

- Decision-makers and stakeholders work mostly together, aiming to find common and mutually acceptable solutions
- Decision-makers and stakeholders partly work together, but not always, in order to find common solutions
- Very little collaborative approaches between decision-makers and stakeholders towards finding common solutions
- Not sure
- Other: ____________________

13. Please explain your answer above.

Learning together (social-learning)

14. Are you or have you ever been part of any forum/platform with multiple and diverse stakeholders that deliberate and reflect on the governance and management of the Knysna Estuary?

Mark only one oval.

- Yes, I actively participate in such a forum/platform
- Yes, I used to participate in such a forum/platform
- No, I have never been part of or participated in such a forum/platform, but I am aware of such forums/platforms
- No, I have never been part of such a forum/platform and I am not aware of any such forums/platforms
- Other: ____________________

15. (Optional) Please indicate in which forums/platforms you participate that deliberate and reflect on the governance and management of the Knysna Estuary

16. Tell us if you if over the past five years you heard, read or experienced something regarding the Knysna Estuary that changed your viewpoints or opinions on the governance or management of the estuary?

Complexity thinking

17. Which statement below do you think best describes the information needs for effectively governing and managing the Knysna Estuary? You may feel that you agree with more than one statement below, but please just indicate the one you feel BEST describes the current situation, or alternatively provide your own description.

Mark only one oval.

- There is not enough information on the Knysna Estuary to manage it effectively. There should be a concerted effort to do targeted research in order to learn how the system works, and then this knowledge should be used to effectively and efficiently manage the estuary
- Barring a few knowledge gaps, we already know enough of the Knysna Estuary to manage it efficiently and in a predictable manner – we know what should be done, it is just a matter of getting on with doing it
- Although we have some understanding of the social and ecological aspects of the Knysna Estuary, these are intertwined and inter-connected, making it hard to fully predict how the ecology and people's use of and impact on the estuary will change in future. As such, we will have to accept that we need to learn as we go along managing the system, and we will invariably make some mistakes along the learning journey
- Other: ____________________

18. Please motivate your answer above

General Comments

19. What is your dream for the Knysna Estuary in the next 10 years?

20. Feel free to make any further comments on the governance of the Knysna Estuary (optional)

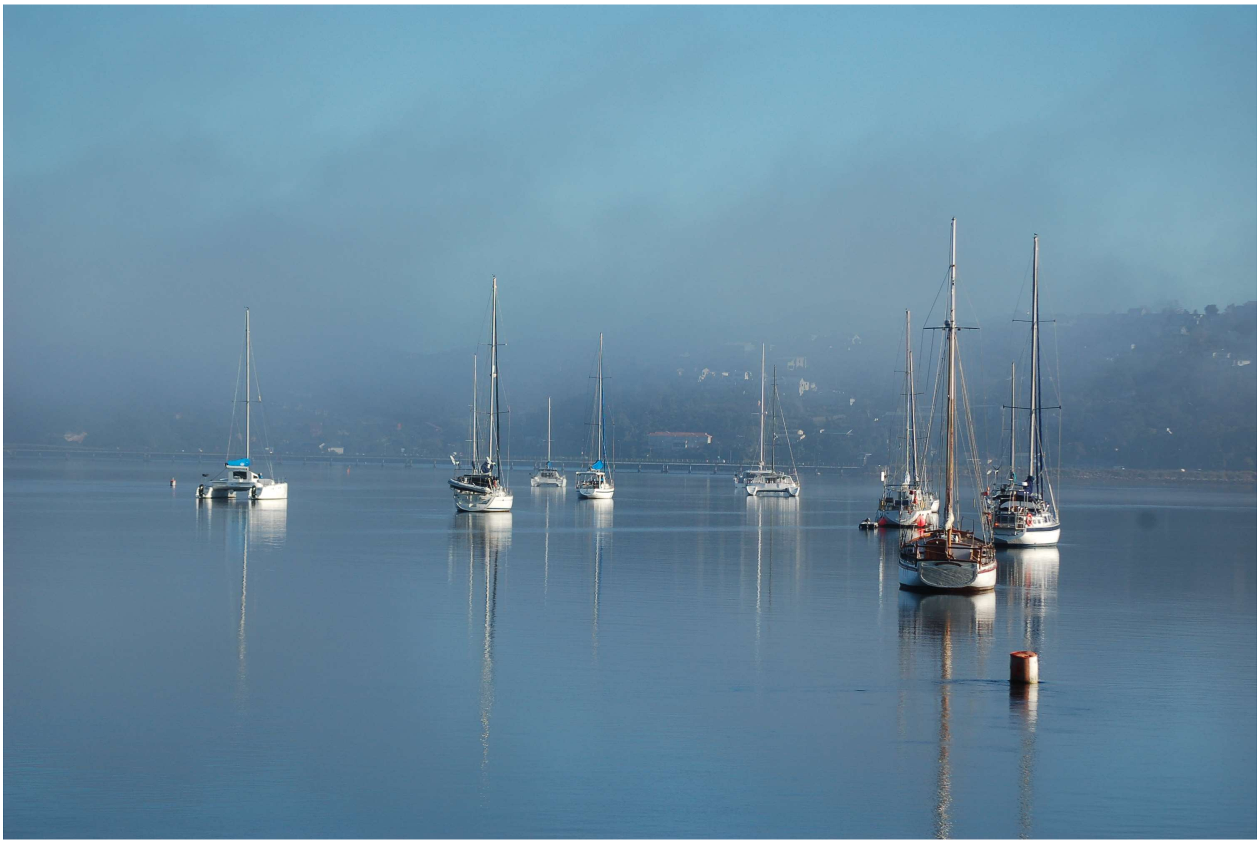

This content is neither created nor endorsed by Google.

Google Forms
