# Supplementary material for: Co-Producing Narratives and Indicators as Catalysts for Adaptive Governance of a Common-Pool Resource within a Protected Area
Source: Environ Manage. 2023 Sep 23;72(6):1111–27. doi: 10.1007/s00267-023-01884-z (PMC10570219; doi:10.1007/s00267-023-01884-z)
Supplement: Supplementary file 3 — Supplement 3 [file 267_2023_1884_MOESM3_ESM.pdf]

Knysna Estuary Post-dialogue Stakeholder Survey

Dear Stakeholder,

You were previously invited to complete an anonymous online questionnaire (June 2021) and/or participated in an online discussion session (10 June 2021) on the above topic. About 30 different groups of stakeholders have been involved in this process to date.

Based on a combination of theory and your inputs to date, we propose eight indicators to promote adaptive governance of Knysna Estuary. The indicators are based on four principles: the need for multi-level governance; collaborative governance; social learning and complexity thinking. These principles were unpacked and discussed in the online questionnaire and discussion.

We would like your input once more to help finalise the indicators. As previously discussed, we believe that these indicators will help to guide collaborative arrangement for the future management of Knysna Estuary, including through the Knysna Estuary Management Plan which needs to be developed during the course of 2022. It is therefore important to get your input and I would appreciate it if you could make the time available to complete one more online survey.

Although many of you have busy schedules, we are keen to provide you with an update on this study sooner rather than later. Attached please find a background document that provides a summary of the process and progress to date to assist in refreshing your thoughts. The survey will remain open until the end of February 2022, which allows you the flexibility to do it at a time that suits you best. If you cannot do it now, it may be useful to schedule some time in your diary before the end of February 2022. I will also send a reminder in two weeks' time.

The survey will take approximately 30 minutes to complete and it will be useful to read the attached background document before doing the survey. Please note that you have to complete and submit the survey in one go – there is not an option to save your input and return to the survey at a later time. You can access the survey here: (survey link removed)

Thank you for your support.

Regards,

(Name removed)
Park Manager: Knysna
South African National Parks

Knysna Estuary Governance

For each of the indicators below, please score the current governance of the Knysna Estuary as you perceive it. The 5-point scale ranges from "very low" to "very high", with descriptions of "very low" (1), "intermediate" (3) and "very high" (5) provided. Feel free to use in-between scoring options (i.e. low = 2 or high = 4) if more appropriate.

Please also
- share your motivation for your score (the motivation is often more valuable than the score),
- indicate how appropriate you consider the indicator for the governance of Knysna Estuary
- share any suggestions to make the indicator more relevant for the case of Knysna Estuary.

The survey should take about 20-30 minutes. Please note the pages of the survey will not save if you lose Internet connection during the survey or halt the survey before completing it. Your survey is only saved once you click “submit” on the last page.

Please read the attached background information document which will give you a summary of the process and outcomes thus far.

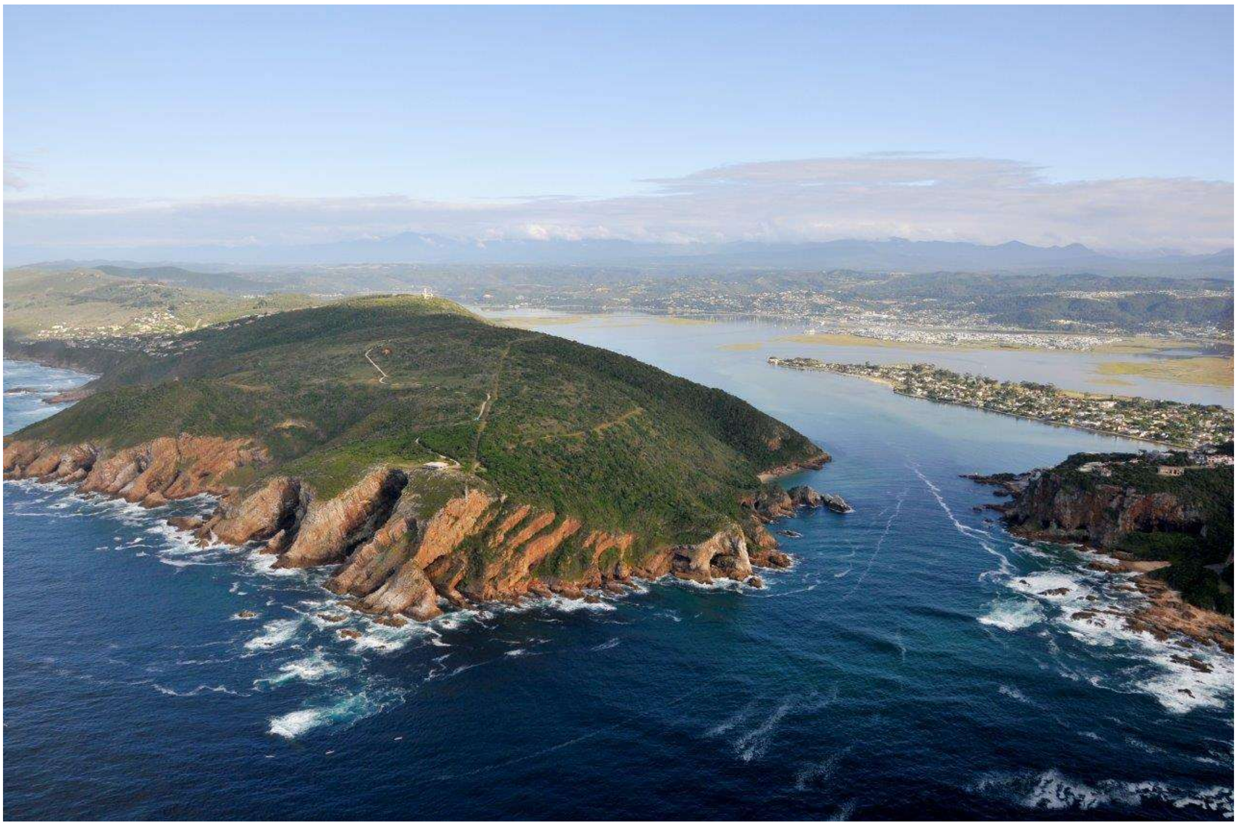

Multi-level governance

Please score Indicators 1 and 2 that reflect on current levels of multi-level governance of the Knysna Estuary.

1. Indicator 1: Decision-makers and those with supporting roles are clearly defined and represent local, provincial and national levels. There are also many types of organisations, such as government, NGO and private, involved.

Mark only one oval.

- 1) Score very low: It is unclear who all the decision-makers are
- 2) Score low
- 3) Score intermediate: It is clear who decision-makers are and who plays supporting roles
- 4) Score good
- 5) Score very good: Decision-makers are clearly identified, AND span local, provincial and national scales as well as diverse organisational forms (government, NGO, private)

2. Please motivate/explain your choice above

____

____

____

____

____

3. Do you consider Indicator 1 appropriate for assessing governance within the context of the Knysna Estuary?

Mark only one oval.

- Very inappropriate indicator
- Inappropriate indicator
- Appropriate indicator
- Very appropriate indicator
- Unsure

4. (Optional). Please motivate your answer above and feel free to share if you have any ideas on how to improve Indicator 1 to make it more appropriate as an indicator for reflecting on the governance performance of Knysna Estuary.

5. Indicator 2: Decision-makers and those with supporting roles are connected, take each other into account and are capable of resolving conflicts

Mark only one oval.

- 1) Score very low: Decision-making centres operate mostly in silos
- 2) Score low
- 3) Score intermediate: Decision-makers have regular meetings and discuss their mandates, initiatives and intentions for the estuary and its stakeholders
- 4) Score high
- 5) Score very high: Decision-makers meet regularly AND there are conflict resolution mechanisms AND coordination between decision-makers are budgeted for (e.g. costs for consultation, reaching agreement and enforcing these)

6. Please motivate/explain your choice above.

7. Do you consider Indicator 2 appropriate for assessing governance within the context of the Knysna Estuary?

Mark only one oval.

- Very inappropriate indicator
- Inappropriate indicator
- Appropriate indicator
- Very appropriate indicator
- Unsure

8. (Optional). Please indicate if you have ideas on how to improve Indicator 2 to make it easier and/or more meaningful as an indicator for reflecting on governance performance.

Collaborative governance

Please score Indicators 3 and 4 as part of your reflection on current levels of collaborative governance of the Knysna Estuary.

9. Indicator 3: Decision-makers have a shared understanding of management challenges, agree on the need for collaboration, and cooperatively contribute towards a shared vision for the estuary.

Mark only one oval.

- 1) Score very low: There is little evidence of a shared understanding of management challenges and complementary roles and responsibilities of decision-makers
- 2) Score low
- 3) Score intermediate: There is a shared understanding of key management challenges and it is accepted that no single party has all the answers. It is understood that lack of collaboration will have negative consequences for all. The majority of stakeholders buy in to a vision for Knysna Estuary and align their actions to contribute towards achieving this vision
- 4) Score high
- 5) Score very high: Collaboration includes pooling of resources to achieve the collective vision; decisions on key issues are made through consensus; broad buy-in reinforces compliance with rules (e.g. on bait collecting limits, fishing bag limits, wake speeds, allowable effluent quality)

10. Please motivate/explain your choice above.

11. Do you consider Indicator 3 appropriate for assessing governance within the context of the Knysna Estuary?

Mark only one oval.

- Very inappropriate indicator
- Inappropriate indicator
- Appropriate indicator
- Very appropriate indicator
- Unsure

12. (Optional). Please indicate if you have ideas on how to improve Indicator 3 to make it easier and/or more meaningful as an indicator for reflecting on governance performance.

____
____
____
____
____

13. Indicator 4: A leadership body actively coordinates collaborative actions towards achieving the collective vision for Knysna Estuary

Mark only one oval.

- 1) Score very low: There is no clear and generally accepted leadership body to coordinate collaboration towards achieving the vision
- 2) Score low
- 3) Score intermediate: There is a designated and accepted leadership body and some coordination of collaboration
- 4) Score high
- 5) Score very high: Clear leadership has emerged to effectively harness different roles, functions and mandates in a complementary way. Relationships between the majority of decision makers and stakeholders is described as trusting. Every stakeholder feel that they can make a positive contribution

14. Please motivate/explain your choice above.

15. Do you consider Indicator 4 appropriate for assessing governance within the context of the Knysna Estuary?

Mark only one oval.

- Very inappropriate indicator
- Inappropriate indicator
- Appropriate indicator
- Very appropriate indicator
- Unsure

16. (Optional). Please indicate if you have ideas on how to improve Indicator 4 to make it easier and/or more meaningful as an indicator for reflecting on governance performance.

Social Learning

Please score Indicators 5 and 6 as part of your reflection on current levels of social learning in the governance of the Knysna Estuary.

17. Indicator 5: Formal social learning spaces are resourced and enable deliberate and sustained learning among multiple and diverse stakeholders

Mark only one oval.

- 1) Score very low: There are no clear social learning spaces for sustained interaction among diverse stakeholders. Existing forums have narrow interests and do not facilitate co-learning around social-ecological issues
- 2) Score low
- 3) Score intermediate: There is one or more official social learning space open to all stakeholders. Facilitation and resources enable meaningful engagement and repeat meetings
- 4) Score high
- 5) Score very high: There is widespread participation of a diversity of stakeholders in social learning spaces. Participants feel that they can contribute towards making a difference and are comfortable sharing their own expectations and uncertainties

18. Please motivate/explain your choice above.

19. Do you consider Indicator 5 appropriate for assessing governance within the context of the Knysna Estuary?

Mark only one oval.

- Very inappropriate indicator
- Inappropriate indicator
- Appropriate indicator
- Very appropriate indicator
- Unsure

20. (Optional). Please indicate if you have ideas on how to improve Indicator 5 to make it easier and/or more meaningful as an indicator for reflecting on governance performance.

21. Indicator 6: Social learning contributes to integration of diverse forms of knowledge and the uptake of new knowledge in policy and decision making

Mark only one oval.

- 1) Score very low: No or limited participation in social learning opportunities and, as a result, no meaningful change
- 2) Score low
- 3) Score intermediate: Enthusiastic and diverse participation in social learning opportunities leads to widespread diffusion of new information and promotes shared understanding of the estuary and its management challenges
- 4) Score high
- 5) Score very high: Enthusiastic and diverse participation in social learning results in behaviour change. There are feedbacks between social learning and policy processes. The value being created through social learning is clearly visible.

22. Please motivate/explain your choice above.

23. Do you consider Indicator 6 appropriate for assessing governance within the context of the Knysna Estuary?

Mark only one oval.

- Very inappropriate indicator
- Inappropriate indicator
- Appropriate indicator
- Very appropriate indicator
- Unsure

24. (Optional). Please indicate if you have ideas on how to improve Indicator 6 to make it easier and/or more meaningful as an indicator for reflecting on governance performance.

Complexity Thinking

Please score Indicators 7 and 8 as part of your reflection on current levels of complexity thinking in the governance of the Knysna Estuary.

25. Indicator 7: A holistic perspective underpins mental models of stakeholders (beliefs and assumptions about how the estuary works) and decision-making about Knysna Estuary

Mark only one oval.

- ( ) 1) Score very low: There is no explicit model/diagram/representation that depicts Knysna Estuary and its catchment as a complex social-ecological system
- ( ) 2) Score low
- ( ) 3) Score intermediate: Various approaches are readily used to map the mental models of stakeholders and explore the interrelatedness between social and ecological components of the system
- ( ) 4) Score high
- ( ) 5) Score very high: In addition to the above, key feedbacks between social and ecological components are identified AND the ways in which changes to the system may influence the services/benefits derived from the estuary are explored

26. Please motivate/explain your choice above.

____

____

____

____

____

27. Do you consider Indicator 7 appropriate for assessing governance within the context of the Knysna Estuary?

Mark only one oval.

- ( ) Very inappropriate indicator
- ( ) Inappropriate indicator
- ( ) Appropriate indicator
- ( ) Very appropriate indicator
- ( ) Unsure

28. (Optional). Please indicate if you have ideas on how to improve Indicator 7 to make it easier and/or more meaningful as an indicator for reflecting on governance performance.

29. Indicator 8: Management of Knysna Estuary is informed by monitoring and research dealing with the interactions between natural and social systems to advance the sustainability of the overall system

Mark only one oval.

- 1) Score very low: Research on Knysna Estuary is largely disciplinary or reductionist (i.e. based on single disciplines and reduced to single/limited variables and sub-components of the system)
- 2) Score low
- 3) Score intermediate: Disciplinary research is complemented by inter- and trans-disciplinary research that treats the whole Knysna Estuary as the "unit of analysis". It considers both ecological and social variables as well as variables that describe the connections between these
- 4) Score high
- 5) Score very high: A vibrant research and monitoring programme promotes understanding of the whole social-ecological system and key feedbacks / relationships between components. Management acknowledges that the system is unpredictable and carries out frequent revisions of, and adaptations to, management actions and plans. These adaptations reflect changing contexts and new knowledge

30. Please motivate/explain your choice above.

31. Do you consider Indicator 8 appropriate for assessing governance within the context of the Knysna Estuary?

Mark only one oval.

- Very inappropriate indicator
- Inappropriate indicator
- Appropriate indicator
- Very appropriate indicator
- Unsure

32. (Optional). Please indicate if you have ideas on how to improve Indicator 8 to make it easier and/or more meaningful as an indicator for reflecting on governance performance.

In closing

Many thanks for your investment of time and energy into this process of co-learning and improving our collective understanding of the estuary governance. In closing, please answer the last three questions

33. How do you feel about this whole process?

Mark only one oval.

- Disappointed
- Concerned
- Happy
- Excited

34. Do you want to stay involved in the process going forward or do you feel you have invested enough time in the process for now?

Mark only one oval.

- ( ) I would please like to stay involved
- ( ) I have invested enough time in the process for know
- ( ) Other: ____________________

35. If there is anything else you would like to mention, please feel free to do so in the space provided

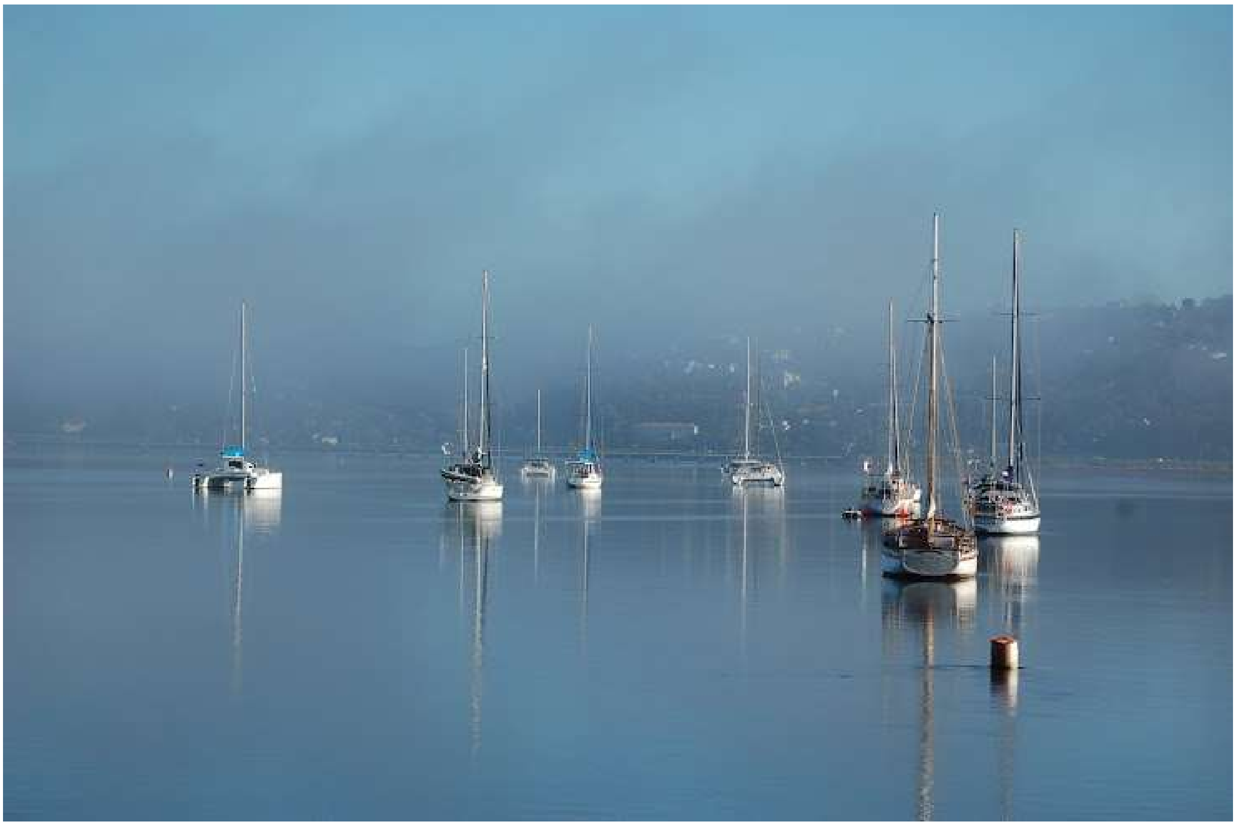

____
____
____
____
____

This content is neither created nor endorsed by Google.

Google Forms
